# Switching between Local and Global Aromaticity in a Conjugated Macrocycle for High‐Performance Organic Sodium‐Ion Battery Anodes

**DOI:** 10.1002/anie.202003386

**Published:** 2020-05-27

**Authors:** Simon Eder, Dong‐Joo Yoo, Wojciech Nogala, Matthias Pletzer, Alejandro Santana Bonilla, Andrew J. P. White, Kim E. Jelfs, Martin Heeney, Jang Wook Choi, Florian Glöcklhofer

**Affiliations:** ^1^ Department of Chemistry and Centre for Processable Electronics Imperial College London Molecular Sciences Research Hub 80 Wood Lane London W12 0BZ UK; ^2^ School of Chemical and Biological Engineering and Institute of Chemical Processes Seoul National University 1 Gwanak-ro, Gwanak-gu Seoul 08826 Republic of Korea; ^3^ Institute of Physical Chemistry Polish Academy of Sciences Kasprzaka 44/52 01-224 Warsaw Poland

**Keywords:** aromaticity, hydrocarbons, macrocycles, organic batteries, voids

## Abstract

Aromatic organic compounds can be used as electrode materials in rechargeable batteries and are expected to advance the development of both anode and cathode materials for sodium‐ion batteries (SIBs). However, most aromatic organic compounds assessed as anode materials in SIBs to date exhibit significant degradation issues under fast‐charge/discharge conditions and unsatisfying long‐term cycling performance. Now, a molecular design concept is presented for improving the stability of organic compounds for battery electrodes. The molecular design of the investigated compound, [2.2.2.2]paracyclophane‐1,9,17,25‐tetraene (PCT), can stabilize the neutral state by local aromaticity and the doubly reduced state by global aromaticity, resulting in an anode material with extraordinarily stable cycling performance and outstanding performance under fast‐charge/discharge conditions, demonstrating an exciting new path for the development of electrode materials for SIBs and other types of batteries.

## Graphical abstract

**Aromatic stabilization of both states**: A molecular design concept for highly stable organic battery electrode materials is presented using the conjugated macrocycle [2.2.2.2]paracyclophane‐1,9,17,25‐tetraene (PCT). The compound can stabilize both the neutral and reduced state by aromaticity, enabling batteries with extraordinarily stable cycling performance and outstanding performance under fast‐charge/discharge conditions.

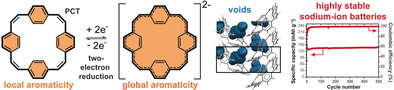

## Introduction

Aromatic organic compounds hold great promise for becoming the next generation of battery electrode materials owing to their low‐cost, environmentally benign, and recyclable nature.^1^ Although lithium‐ion batteries (LIBs) have been greatly successful for various applications, next‐generation materials are desirable to reduce the dependence on toxic heavy metals and lithium as well as to increase the freedom in structure and property tuning.^2^ Sodium‐ion batteries (SIBs) are a much praised alternative to LIBs, but the anode material conventionally used for LIBs, graphite, is inactive for SIBs,^3^ which is due to the thermodynamically unfavorable insertion of sodium ions. To date, the small group of aromatic organic compounds found to be suitable as SIB anode materials largely consists of sodium carboxylates.^4^ While promising specific capacities were achieved with these compounds, most of them suffered from significant degradation issues when tested under fast‐charge/discharge conditions or for long‐term cycling. Fundamentally new concepts are needed for solving these issues and for designing stable, high‐performance organic SIB anode materials.

Recent fundamental studies of conjugated macrocycles indicate that global (anti)aromaticity effects in organic compounds can lead to promising redox properties for battery applications.^5^ Shinokubo et al. investigated a concept that employed global aromaticity to stabilize the doubly reduced or oxidized state of a conjugated macrocycle in order to achieve good redox properties for applications as electrode material in LIBs.^6^ The porphyrinoid, which they used, can switch between an antiaromatic neutral state, featuring a macrocyclic conjugated system of [4*n*] π‐electrons, and an aromatic doubly reduced (or oxidized) state of [4*n*+2] π‐electrons, obeying Hückel's rule. The charged states of the porphyrinoid were stabilized effectively by the global aromaticity, but the concept suffers from the inherent destabilizing effect of the global antiaromaticity in the neutral state. Generally, antiaromatic compounds lack meaningful practical applications owing to their inherent instability. The porphyrinoid used by Shinokubo et al. required steric protection by bulky substituents to counterbalance the destabilizing effect and achieve sufficient stability for testing in batteries.

**Figure 1:**
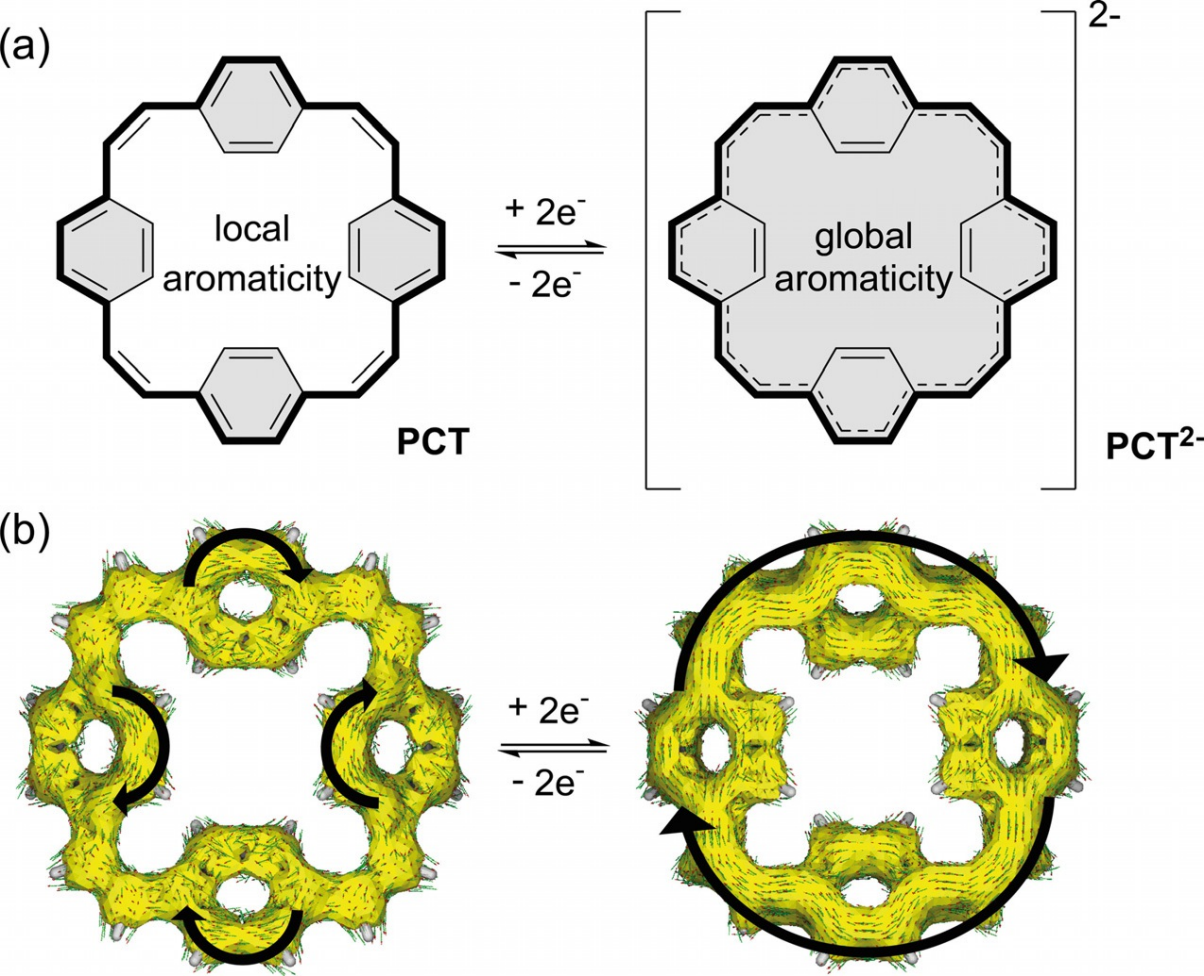
a) Molecular structure of [2.2.2.2]paracyclophane‐1,9,17,25‐tetraene (PCT, left) and the corresponding dianion (PCT^2−^, right); bold bonds indicate the [24]annulene substructure with [4*n*] π‐electrons in the neutral state and [4*n*+2] π‐electrons in the doubly reduced state; shaded areas highlight aromaticity. b) Anisotropy of the induced current density (ACID) plots, which allow visualization of electronic delocalization, of PCT (left) and PCT^2−^ (right) at an isovalue of 0.04; large arrows indicate the direction of the small current density vectors, which show diatropic (clockwise, aromatic) ring current in both the neutral and the doubly reduced state.

Herein we explore the concept of switching between a locally aromatic neutral state (instead of a globally antiaromatic neutral state) and a globally aromatic doubly reduced state, overcoming the inherent issues of antiaromaticity and providing an exciting new design concept for organic electrode materials. Instead of bulky substituents (as used for stabilizing the porphyrinoid), we introduced vinylene bridges along the [4*n*] π‐electron system of an antiaromatic conjugated macrocycle known as [24]annulene (Figure 1 a left; [24]annulene substructure indicated by bold bonds), creating locally aromatic phenylene units with [4*n*+2] π‐electrons that we expected to dominate the structure. Surprisingly, although the conjugated macrocycle resulting from this design process, [2.2.2.2]paracyclophane‐1,9,17,25‐tetraene (PCT), was subject to intense fundamental studies in the 1970s and 1980s,^7^ our study is the first to explore the concept of switching between local and global aromaticity and to investigate the benefits of the macrocyclic structure for applications as electrode material.

## Results and Discussion

PCT was obtained in a single step from low‐cost starting materials terephthalaldehyde and *p*‐xylylenebis(triphenylphosphonium bromide) by a Wittig reaction (see the Supporting Information for details). Purification of the crude product by preparative gel permeation chromatography (GPC) afforded PCT as a pure, bright yellow powder in yields of 13 %. As a scalable alternative purification method, sublimation instead of GPC proved to be feasible at moderately reduced pressure (approx. 0.4 mbar) and elevated temperature (240 °C). Similar yields of 11 % were obtained by sublimation, with no differences in purity according to ^1^H NMR measurements (Supporting Information, Figures S1 and S2). Such a simple, low‐cost synthesis and purification is rarely achieved for conjugated macrocycles but is important to facilitate preparation on the scale required for battery electrodes.

As a first step in assessing our molecular design, we investigated its ability to stabilize the neutral state of PCT by determining the thermal properties of the compound. Thermogravimetric analysis (TGA) revealed high thermal stability, with the decomposition starting at approx. 290 °C and a 5 % mass loss at 317 °C (Supporting Information, Figure S5). Differential scanning calorimetry (DSC) showed a melting point at 245 °C (Supporting Information, Figure S6). Neither in the solid state, nor in solution, were any stability issues observed by ^1^H NMR measurements. In contrast, [24]annulene (obtained in three steps in an overall yield of approx. 0.1 %) was reported to decompose on attempted melting point determination and almost fully decomposed at room temperature within 24 hours.^8^ The high stability of PCT compared to its parent compound [24]annulene confirmed the intended effect of creating locally aromatic units along the [4*n*] π‐electron system on the stability of the compound.

To investigate the effect of the molecular design on the ring currents, we carried out anisotropy of the induced current density (ACID) calculations^9^ as a next step. The ACID plot of neutral PCT shows slightly disturbed diatropic (aromatic, clockwise) currents on the four phenylene units (Figure 1 b left). No global ring current was observed along the [24]annulene substructure. Hence, according to these calculations, neutral PCT can indeed be regarded as composed of locally aromatic phenylene units with [4*n*+2] π‐electrons connected by vinylene units, with no significant antiaromatic contribution from the global [4*n*] π‐electron system of the [24]annulene substructure. This observation is in accordance with the experimentally determined ^1^H NMR signals at 7.32 ppm (phenylene) and 6.42 ppm (vinylene). Furthermore, low‐temperature ^1^H NMR measurements showed no indication of antiaromatic character on cooling to 193 K (Supporting Information, Figure S4).

Twofold reduction of PCT to the corresponding dianion PCT^2−^ (Figure 1 a right) drastically changes the ring current flow. The ACID plot of PCT^2−^ shows a strong diatropic ring current along the [24]annulene substructure (Figure 1 b right), indicating that the two additional electrons delocalize over the macrocyclic substructure and create a globally aromatic [4*n*+2] π‐electron system that obeys Hückel's rule. The diatropic ring current mainly flows along the perimeter of the macrocycle, confirming conclusions previously drawn from ^1^H NMR measurements of the dianion, which experimentally indicated its globally aromatic nature.7d No local ring currents were observed in the ACID plots of PCT^2−^. The plots effectively visualize the transition from a locally aromatic neutral state to a globally aromatic doubly reduced state. Upon further reduction, the calculations predict a strong paratropic (antiaromatic, counter‐clockwise) ring current in the tetraanion PCT^4−^, followed by recovery of the diatropic ring current in the hexaanion PCT^6−^ (Supporting Information, Figure S11). Similar effects are predicted for the corresponding cations (Supporting Information, Figure S12).

To experimentally investigate the reduction of PCT and confirm that a global [4*n*+2] π‐electron system is formed, we conducted cyclic voltammetry (CV) measurements in solution. Previous CV measurements on a hanging mercury drop electrode indicated that the reduction of PCT in DMF is a reversible two‐electron process.7c This conclusion was drawn from the small difference of the cathodic and anodic peak potentials (Δ*E_p_*) of 30 mV, which is below the thermodynamic limit for a one‐electron process (57 mV at 25 °C). Although we found the reduction to be chemically and practically reversible (Supporting Information, Figure S13, first 30 cycles), it was not thermodynamically reversible on our glassy carbon electrode (Δ*E_p_*=61 mV). This renders confirmation of the reduction stoichiometry (that is, the number of electrons transferred per molecule) from Δ*E_p_* or the slope of steady‐state voltammograms difficult (also as these parameters are influenced by electrode process kinetics and Ohmic drop). Therefore, we decided to follow a different approach to confirm the stoichiometry and prepared PCT solutions of known concentrations in 0.1 m NBu_4_PF_6_ in *N*,*N*‐dimethylformamide (DMF), propylene carbonate (PC) and 1,2‐dichloroethane (DCE) for CV using a platinum disc ultramicroelectrode (UME) of 25 μm diameter (Supporting Information, Figure S14). Based on the diffusion limited current of the reduction in these measurements, we estimated the diffusion coefficients of PCT in the different solvents, assuming a two‐electron stoichiometry (see the Supporting Information for details). The diffusion coefficients were then used to estimate the hydrodynamic radii of PCT (0.51 nm in DMF and PC, 0.39 nm in DCE), which were in good agreement with the optimized (non‐spherical, cylinder‐like) geometry of PCT (radius: ca. 0.65 nm, height: ca. 0.25 nm; Supporting Information, Figure S9), confirming the two‐electron mechanism of the reduction and the formation of a global [4*n*+2] π‐electron system in all tested solvents. Like the reduction, the oxidation of PCT was found to be a two‐electron process in all three solvents (Supporting Information, Figure S16), but the oxidation was chemically reversible only in DCE (with an estimated redox potential of the PCT/PCT^2+^ couple of 0.77 V vs. ferrocene/ferrocene^+^ (Fc/Fc^+^), see the Supporting Information for details).

**Figure 2:**
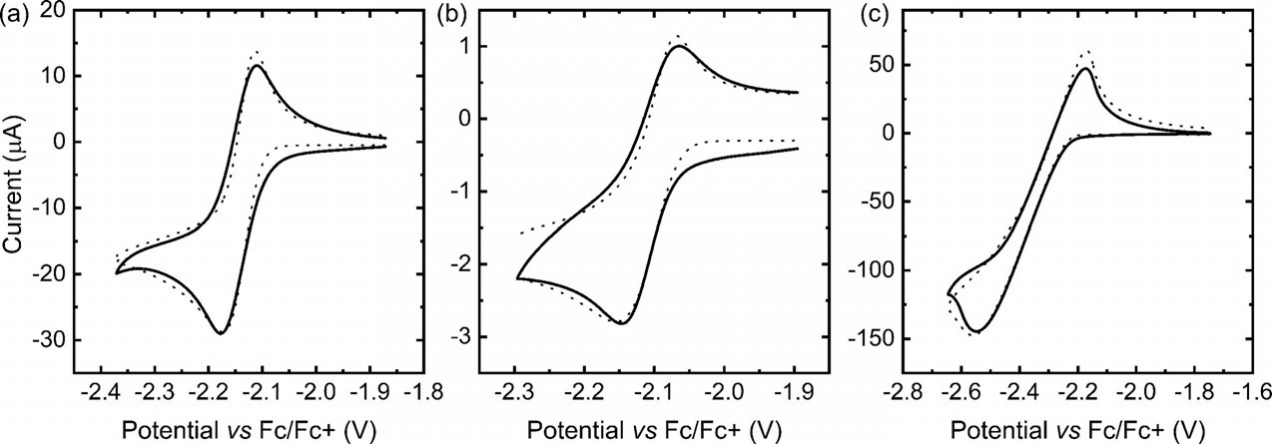
Cyclic voltammograms of PCT in a) DMF (scan rate *v*=10 mV s^−1^, PCT concentration *c*=7.25 mm), b) PC (*v*=100 mV s^−1^, *c*=0.32 mm), and c) DCE (*v*=100 mV s^−1^, *c=*12.8 mm) recorded on platinum disc electrodes of 2 mm diameter (solid lines). Supporting electrolyte: 0.1 m NBu_4_PF_6_. Simulated cyclic voltammograms (dotted lines) for fitting of kinetic parameters. Diffusion coefficients, concentrations, and fitted kinetic parameters are listed in the Supporting Information, Table S2. Other fitted parameters: a) uncompensated resistance (*R*_u_): 100 Ω, capacitance of the electric double layer at the working electrode‐electrolyte interface (*C*_dl_): 50 μF; b) *R*_u_=100 Ω, *C*_dl_=3 μF; c) *R*_u_=1000 Ω, *C*_dl_=10 μF. Temperature: 293.2 K.

For estimating the kinetic parameters of the reduction, we recorded cyclic voltammograms of the same PCT solutions on a larger platinum disc electrode of 2 mm diameter and fitted simulated voltammograms to the measured voltammograms (Figure 2). This allowed us to estimate standard electron transfer rate constants (*k*_0_) of 1.8×10^−3^ cm s^−1^ (DMF and PC) and 1.0×10^−3^ cm s^−1^ (DCE) as well as electron transfer coefficients (α) of 0.17 (DMF), 0.32 (PC) and 0.29 (DCE) (see the Supporting Information for details). The rate constants correspond to thermodynamically quasi‐reversible cases.^10^ The kinetics are faster than the electroreduction of Li^+^ in DMF (*k*_0_=4.7×10^−4^ cm s^−1^),^11^ but slower than thermodynamically reversible cases, for example, ferrocene oxidation in acetonitrile (*k*_0_=8.4 cm s^−1^).^12^

Evaluation of the redox potential of the PCT/PCT^2−^ couple revealed a solvent‐dependent potential as low as −2.29 V vs. Fc/Fc^+^ in DCE. Chemically reversible reduction of aromatic organic compounds at such low potential is very unusual but highly beneficial for application as an anode material in batteries. A low reduction potential increases the difference between the redox potentials of cathode and anode and, hence, enables higher operating voltage in full cells. While we attribute the chemical reversibility of the reduction to the stabilization of PCT^2−^ by global aromaticity/ delocalization of the charges in the macrocycle, the low redox potential is the result of another exciting feature of the molecular design: PCT is a pure hydrocarbon with no heteroatoms or functional groups that would shift the redox potential to higher values by withdrawing electron density from the conjugated π‐electron system. Such functional groups or heteroatoms are usually required to store carrier ions in the reduced form; the molecular design of PCT solves this conundrum. For comparison, the porphyrinoid mentioned in the introduction showed a redox potential of −0.90 V vs. Fc/Fc^+^ for the first reduction and −1.67 V vs. Fc/Fc^+^ for the second reduction (in CH_2_Cl_2_),^6^ which results in a lower operating voltage of the full‐cell (compared to PCT) but also in a voltage profile with an unfavorable slope over a large potential range.

**Figure 3:**
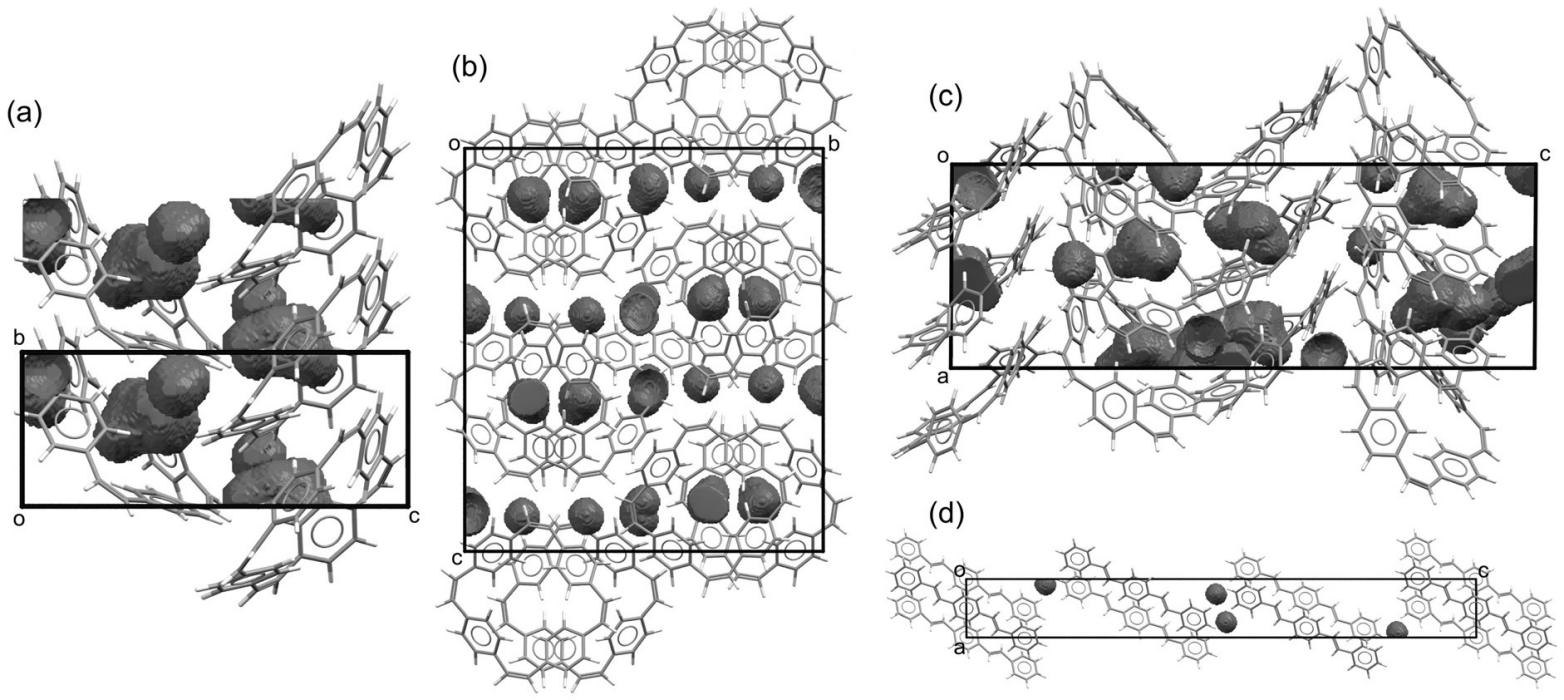
Crystal structures of different PCT polymorphs showing voids large enough to hold sodium ions: a) grown from acetic acid solution (with one of the molecules in the unit cell in orientation A and the other molecule in orientation B, viewed along the *a*‐axis; 5.8 % of the unit cell volume are empty space), b) previously reported crystal structure (viewed along the *a*‐axis; 4.9 %), c) grown by sublimation (viewed along the *b*‐axis; 5.5 %). d) Crystal structure of 1,4‐distyrylbenzene (viewed along the *b*‐axis; 0.8 %).

In contrast to reductions in solution, counterions (sodium ions in SIBs) need to be able to insert into the solid‐state anode material during the reduction (charging) process. To assess the capability of PCT to host sodium ions, we analyzed its solid‐state packing as a next step. X‐ray diffraction (XRD) analysis of single crystals grown from acetic acid solution revealed an extensively disordered structure with two overlapping orientations (termed orientation A and B here) occurring in a ratio of ca. 56:44 (Supporting Information, Figure S17). Interestingly, we found large voids in the crystal packing, which were not occupied by solvent molecules. Assuming that one of the two PCT molecules in the unit cell adopts orientation A and the other molecule orientation B and placing a probe of the radius of Na^+^ (1.02 Å) on a regularly spaced grid (0.1 Å spacing) in this unit cell to identify empty space, we estimated that 5.8 % (66.68 Å^3^) of the unit cell is empty space large enough to hold sodium ions (see Figure 3 a). This equals the volume of 15.0 Na^+^ per unit cell or 7.5 Na^+^ per PCT molecule, easily providing space for the two counterions per molecule expected to insert upon twofold reduction. Assuming an A‐only (B‐only) orientation, the same analysis indicated that 4.1 % (8.1 %) of the unit cell volume can hold sodium ions (Supporting Information, Figure S19). With a probe of the radius of Li^+^ (0.76 Å), these values increased to 15.5 % (AB), 14.1 % (A‐only) and 17.0 % (B‐only; Supporting Information, Figure S20).

However, the XRD pattern of the PCT powder used later for electrode preparation (as obtained after purification by GPC), did not match our single‐crystal data. Luckily, the pattern did match with a polymorph reported in the 1970s (CCDC 12295457b) (Supporting Information, Figure S26a). Analysis of the crystal structure of this polymorph by the method described above revealed similarly large void volumes of 4.9 % (Na^+^; Figure 3 b) and 14.9 % (Li^+^; Supporting Information, Figure S22) of the unit cell. Although the authors reported that the single crystal of this polymorph was obtained from acetic acid solution, powder XRD of ground crystals grown from acetic acid matched well with our newly reported crystal structure (Supporting Information, Figure S26b).

XRD analysis of a single crystal that we obtained by sublimation confirmed that large voids are a general property of solid‐state PCT; void volumes of 5.5 % (Na^+^; Figure 3 c) and 14.9 % (Li^+^; Supporting Information, Figure S25) were found in this polymorph. For comparison, we analyzed the crystal structure of 1,4‐distyrylbenzene (CCDC 921998^13^), a structurally related linear compound. The analysis revealed significantly smaller void volumes of 0.8 % (Na^+^; Figure 3 d) and 3.1 % (Li^+^; Supporting Information, Figure S27), corroborating our theory that the macrocyclic geometry inhibits dense packing and provides voids for counterions, facilitating the intermolecular diffusion of ions and preventing unfavorable volume expansion during the charging process. Furthermore, in all of the polymorphs, the distances between macrocycles were found to be larger than 3.7 Å (Supporting Information, Figures S18, S21, and S24), which was predicted to be the minimum spacing between layers required for sodium insertion (sodiation) in carbon materials.^14^

**Figure 4:**
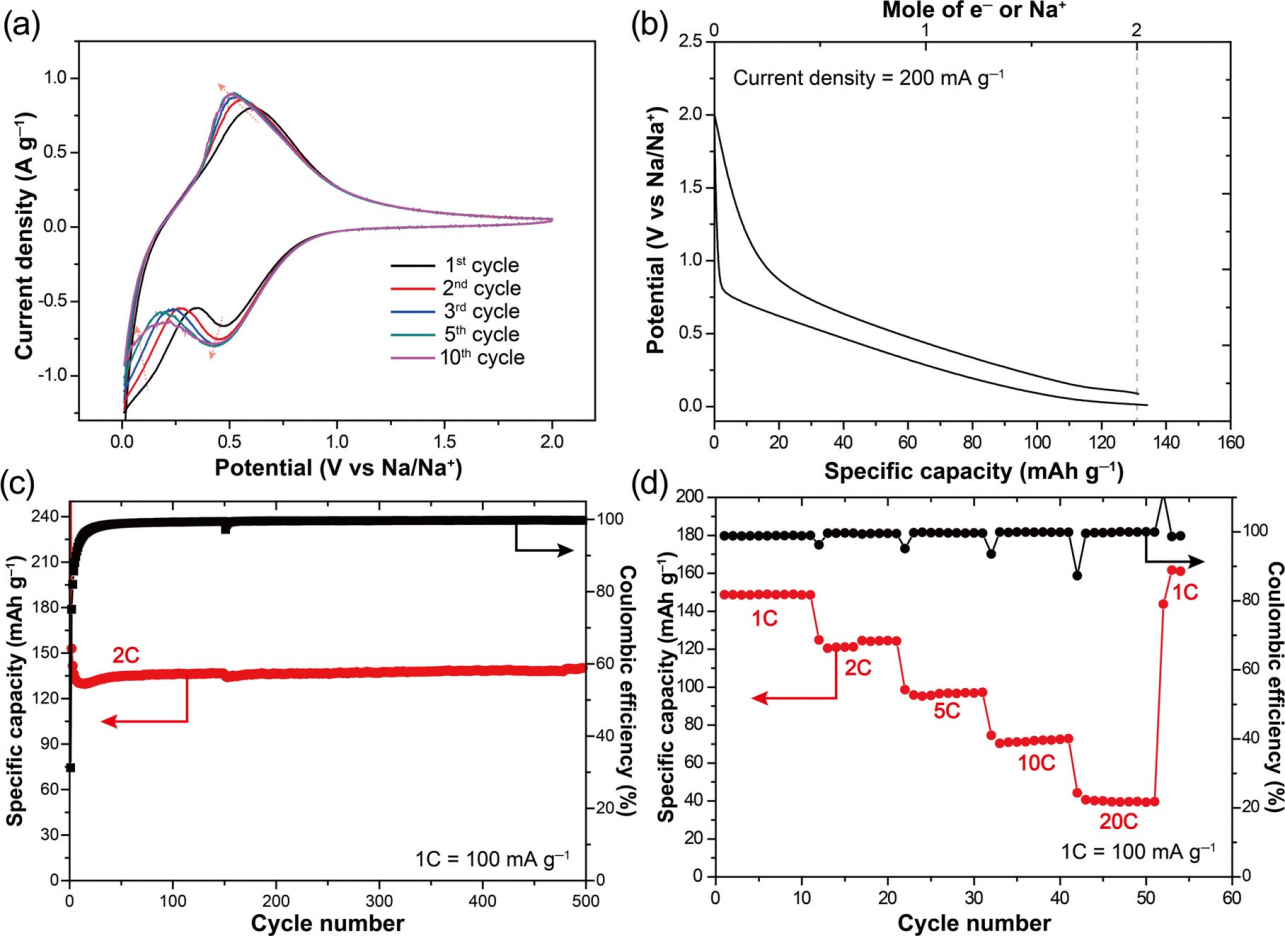
a) Cyclic voltammograms of the PCT electrode in the voltage range of 0.01–2.0 V (scan rate: 1 mV s^−1^). b) Voltage profile of the PCT electrode at a current density of 200 mA g^−1^. c) Cycling performance and d) rate capability test of PCT at various current densities. Black circles in (d) correspond to Coulombic efficiencies.

Given this promising combination of properties, we were excited to finally assess PCT as an anode material in SIBs. As a first step in the assessment, CV was conducted in the range of 0.01–2.0 V at a scan rate of 1 mV s^−1^ to confirm the electrochemical activity of solid‐state PCT in the anode potential range (Figure 4 a). A pair of reduction and oxidation peaks was observed at 0.4 and 0.5 V vs. Na/Na^+^, respectively, and these potentials are similar to those of hard carbons, the most popular anode materials in SIBs, which undergo reduction involving delocalized π orbitals for sodium ion storage.^15^ The peak current density increased during the first few cycles, presumably due to some interfacial activation. From a so‐called *b*‐value analysis (Supporting Information, Figure S28), which is based on the reduction and oxidation peak current densities in CV measurements at different scan rates, we concluded that the sodium ion storage with PCT is mainly diffusion‐controlled, as the *b*‐values of the reduction and oxidation peaks were 0.56 and 0.64, respectively. A subsequent galvanostatic test at a current density of 200 mA g^−1^ (Figure 4 b) showed that PCT exhibits a specific capacity of 133 mAh g^−1^ at this current density, which is close to its theoretical capacity of 131 mAh g^−1^ corresponding to twofold reduction and storage of two Na^+^ ions per PCT molecule. In this galvanostatic test, the voltage monotonically decreased in the range of 0.8–0.01 V, which is in contrast to the voltage profile of hard carbons that show two distinct sodium ion storage regimes (interlayer storage between the graphene layers and adsorption within the micropores).15b The two regimes result in steep and sloppy slopes in the voltage profile, respectively, making the operating voltage of hard carbons less definitive.

To confirm the charge storage mechanism and delocalization of the electrons in the π‐conjugated system of the macrocycles, we conducted ex situ X‐ray photoelectron spectroscopy (XPS) analysis of pristine, sodiated, and desodiated electrodes. In the C 1s branch (Supporting Information, Figure S29a), the C=C bond disappeared upon sodiation and the C−C bond was shifted to a lower energy on account of the reduction of PCT engaging the π orbitals of the conjugated macrocycle.^16^ The sodiation was also clearly reflected in the appearance of a peak at 1072.1 eV in the Na 1s branch (Supporting Information, Figure S29b). Besides the observed sodiation, the formation of the solid electrolyte interphase (SEI) layer was detected in the C 1s and F 1s branches for both the sodiated and desodiated states; the peak of the sodiated electrode at 288.2 eV in the C 1s branch (Supporting Information, Figure S29a) can be assigned to Na_2_CO_3_, whereas the peak at 287.4 eV of the desodiated electrode can be assigned to COOR. In a similar context, NaF was detected as an SEI component at 684.2 eV in F 1s branch (Supporting Information, Figure S29c). After desodiation, the peak corresponding to the C−C bond was mostly restored, although the peak assigned to the C=C bond was not as much, presumably due to the SEI formation.

For evaluating the electrochemical stability of PCT during sodium (de)insertion, we finally conducted cycling and rate capability tests using electrodes with 30 wt % of PCT as the active material. As shown in Figure 4 c, PCT showed extraordinarily stable cycling performance without any capacity fading at all over 500 cycles when measured at 2 C (1 C=100 mA g^−1^), confirming that our molecular design effectively stabilized the electrode in both the neutral and reduced state and that solubility of the compound is not an issue (see also the Supporting Information, Figure S30). As the initial changes in the CV measurements, we attributed the observed gradual initial capacity increase to interfacial activation, but an in‐depth analysis is required to clarify further. Stable cycling performance was also observed when testing electrodes with a high weight content (50 wt %) of PCT (Supporting Information, Figure S31). The rate capability test (Figure 4 d) further revealed outstanding performance under fast‐charge/discharge conditions. PCT exhibited capacity retentions of 81, 64, 48, and 27 % with respect to its initial capacity of 148 mAh g^−1^ at 1 C when the C‐rate was increased to 2 C, 5 C, 10 C, and 20 C, respectively. The corresponding voltage profiles are presented in the Supporting Information, Figure S32. When the C‐rate was returned to 1 C, the capacity was recovered to 161 mAh g^−1^, verifying the very robust nature of PCT under high C‐rates. The higher capacity at 1C than the theoretical capacity of PCT is attributed to some capacity of the conductive agent denka black (50 wt %) used in the electrode (the voltage profiles and capacity retention of denka black are provided in the Supporting Information, Figure S33).

## Conclusion

Our results show that stabilizing the charged state of conjugated macrocycles by global aromaticity is a very effective strategy to obtain high‐performance organic battery electrode materials, if the stability of the neutral state is also considered. Designing macrocycles that are globally aromatic in the charged state in such a way that their neutral state can be stabilized by local aromaticity, as we did on the example of [2.2.2.2]paracyclophane‐1,9,17,25‐tetraene (PCT), can prevent destabilizing global antiaromaticity in the neutral state and result in highly stable organic compounds for battery electrodes. As a result of this molecular design, the compounds can switch between a stable locally aromatic and a stable globally aromatic state and, thus, show excellent redox properties even without introducing functional groups or heteroatoms, as we could demonstrate in cyclic voltammetry (CV) measurements of PCT. Assessment of PCT as an anode material in sodium‐ion batteries (SIBs), where the exceptionally low reduction potential of −2.29 V vs. ferrocene/ferrocene^+^ (Fc/Fc^+^) of the compound is of particular benefit, confirmed that the molecular design concept can afford organic electrode materials with excellent performance under fast‐charge/discharge conditions and without capacity fading over hundreds of cycles. The assessment also revealed that the two‐electron nature of the reduction has a beneficial effect on the voltage profile of the electrode. We can further conclude from our results that the macrocyclic geometry of PCT leads to voids in the solid‐state packing capable of hosting sodium ions, which is considered to facilitate the insertion of ions during the charging process and may further explain the excellent performance of the material as an SIB anode.

The stepwise approach in assessing PCT set out in this work can serve as a template for designing and assessing conjugated macrocycles for battery electrodes, with relatively simple anisotropy of the induced current density (ACID) calculations giving a good indication of the capability of the compounds to switch between local and global aromaticity before experiments are performed.

## Conflict of interest

The authors declare no conflict of interest.

## Supporting information

As a service to our authors and readers, this journal provides supporting information supplied by the authors. Such materials are peer reviewed and may be re‐organized for online delivery, but are not copy‐edited or typeset. Technical support issues arising from supporting information (other than missing files) should be addressed to the authors.

Supplementary
