# Supplementary material for: Switching between Local and Global Aromaticity in a Conjugated Macrocycle for High‐Performance Organic Sodium‐Ion Battery Anodes
Source: Angew Chem Int Ed Engl. 2020 May 27;59(31):12958–64. doi: 10.1002/anie.202003386 (PMC7496320; doi:10.1002/anie.202003386)
Supplement: Supplementary file 1 — Supplementary [file ANIE-59-12958-s001.pdf]

Angewandte Chemie
Eine Zeitschrift der Gesellschaft Deutscher Chemiker

Supporting Information

Switching between Local and Global Aromaticity in a Conjugated Macrocycle for High-Performance Organic Sodium-Ion Battery Anodes

Simon Eder[+], Dong-Joo Yoo[+], Wojciech Nogala, Matthias Pletzer, Alejandro Santana Bonilla, Andrew J. P. White, Kim E. Jelfs, Martin Heeney, Jang Wook Choi, and Florian Glöcklhofer**

anie_202003386_sm_miscellaneous_information.pdf

Supporting Information

Table of Contents

1 Preparation of [2.2.2.2]Paracyclophane-1,9,17,25-tetraene (PCT) 1

2 ^{1}H and ^{13}C NMR spectra 2

3 Thermal analysis 4

4 Absorption and photoluminescence spectroscopy 5

5 Computational analysis 6

6 Cyclic voltammetry (CV) in solution 10

7 Crystallography 13

8 Sodium-ion battery electrode tests and characterization 21

9 References 24

1 Preparation of [2.2.2.2]Paracyclophane-1,9,17,25-tetraene (PCT)

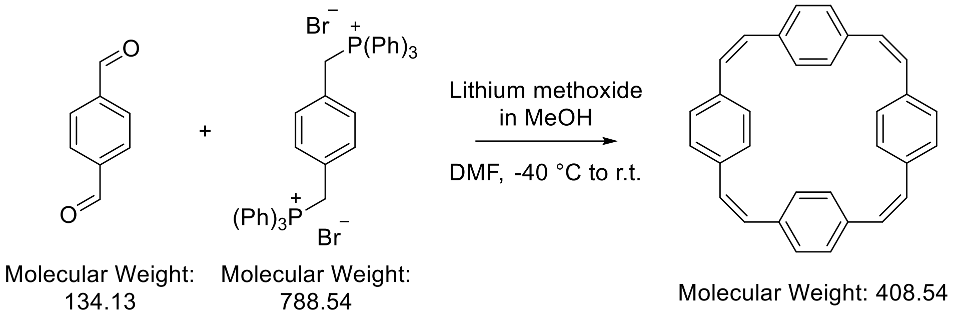

PCT was prepared adapting a previously reported procedure[S1] but replacing silica gel chromatography by filtration over a pad of silica and subsequent preparative gel permeation chromatography (GPC) or sublimation for improved purity:

An oven-dry 500 mL flask was charged with terephthalaldehyde (1.341 g, 10.0 mmol, 1.0 equiv.) and *p*-xylylenebis(triphenylphosphonium bromide) (7.885 g, 10.0 mmol, 1.0 equiv.) and put under nitrogen atmosphere. 300 mL dry degassed *N*,*N*-dimethylformamide (DMF) were added and the resulting white suspension was cooled to -40 °C using an acetonitrile/dry ice cooling bath. Meanwhile, lithium methoxide (LiOMe) (1.14 g, 30.0 mmol, 3.0 equiv.) dissolved in 50 mL dry methanol (MeOH) in an oven-dry 100 mL flask was degassed. The resulting clear solution was added dropwise to the reaction over 9 h using a syringe pump. After complete addition, the reaction was allowed to warm to room temperature and stirred overnight. The resulting bright yellow suspension was poured into 300 mL H_2O and extracted with 3 x 300 mL Et_2O. The combined organic layers were extracted with 2 x 100 mL H_2O and 100 mL brine, dried over $MgSO_4$ and filtered. Evaporation of the solvent *in vacuo* afforded a bright yellow solid, which was suspended in 10 mL CH_2Cl_2 and filtered over a thick pad of silica (to remove triphenylphosphine oxide) using CH_2Cl_2 as eluent. Evaporation of the solvent yielded 943 mg of crude product, which was split into two fractions for purification by gel permeation chromatography (GPC). Each fraction was treated with 5 mL $CHCl_3$ in an ultrasonic bath for 30 min and filtered through a 0.2 µm syringe filter before loading the GPC column with the resulting solution ($CHCl_3$ was used as eluent for the GPC). Evaporation of the combined purified solutions yielded 273 mg **PCT** (0.67 mmol, 13 %) as bright yellow powder. Mp: 245°C (DSC); ^{1}H NMR (400 MHz, $CDCl_3$): δ 7.32 (s, 16H), 6.42 (s, 8H); ^{13}C NMR (100 MHz, $CDCl_3$): δ 136.3, 129.9, 129.0; ^{1}H NMR (400 MHz, CD_2Cl_2, 293 K) δ 7.33 (s, 16H), 6.42 (s, 8H); ^{1}H NMR (400 MHz, CD_2Cl_2, 193 K) δ 7.44 (s, 16H), 6.30 (s, 8H); UV/Vis: λ_{max} 306 nm ($CHCl_3$); HRMS (m/z): $[M+H]^+$ calcd. for $C_{32}H_{24}$, 409.1951; found, 409.1934 (APCI).

As a scalable alternative, the crude product can be purified by sublimation at approx. 0.40 mbar and 240°C, which yielded 223 mg **PCT** (0.54 mmol, 11%) as bright yellow needles (reaction carried out on the same scale as for the purification by GPC).

Purification by silica gel chromatography does not effectively remove oligomeric/polymeric by-products, which may have led to an overestimation of the reaction yield in the previous report.

Purification by preparative GPC was carried out on a LaboACE LC-5060 (Japan Analytical Industry Co., Tokyo, JAPAN) recycling GPC system equipped with a JAIGEL-2HR column and a TOYDAD800-S detector. $CHCl_3$ was used as the eluent at a flow rate of 10 mL min^{-1}.

2 ^{1}H and ^{13}C NMR spectra

NMR spectra were recorded at 400 MHz for ^{1}H and 100 MHz for ^{13}C on a Bruker AV-400.

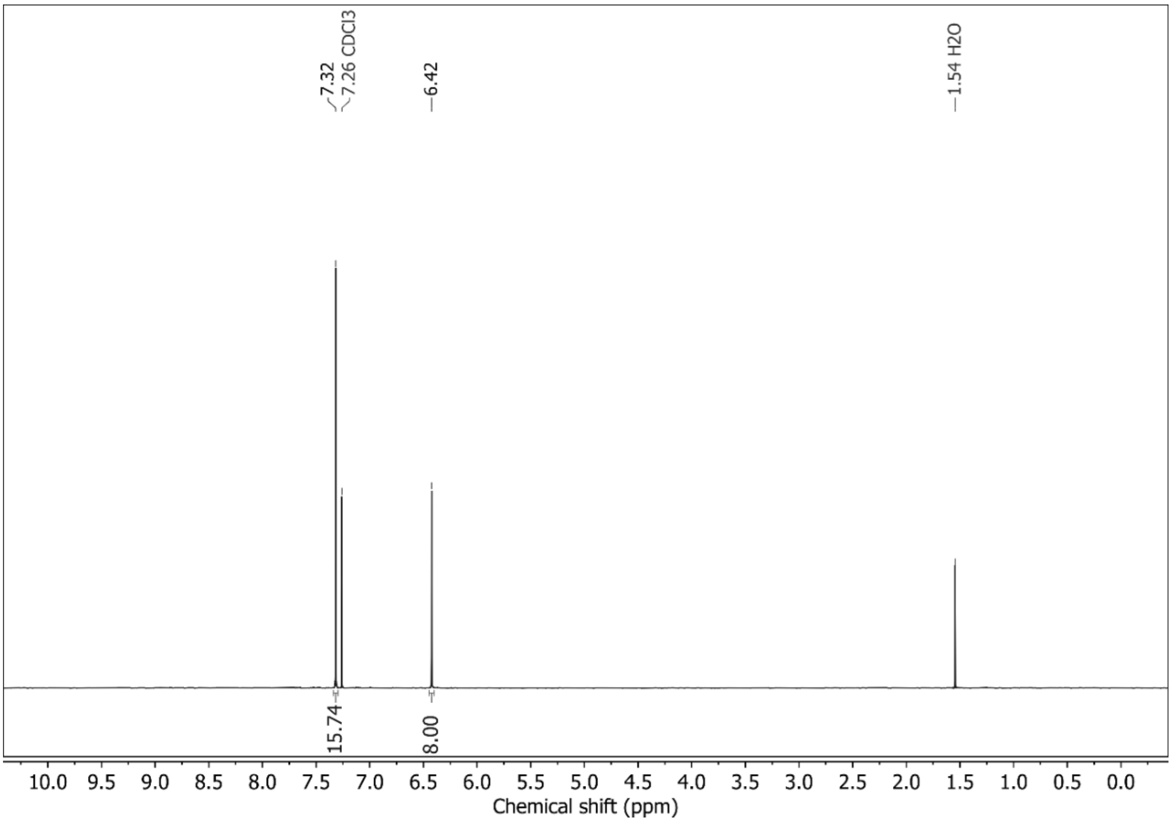

Figure S1. ^{1}H NMR (400 MHz, $CDCl_3$) of **PCT** purified by GPC.

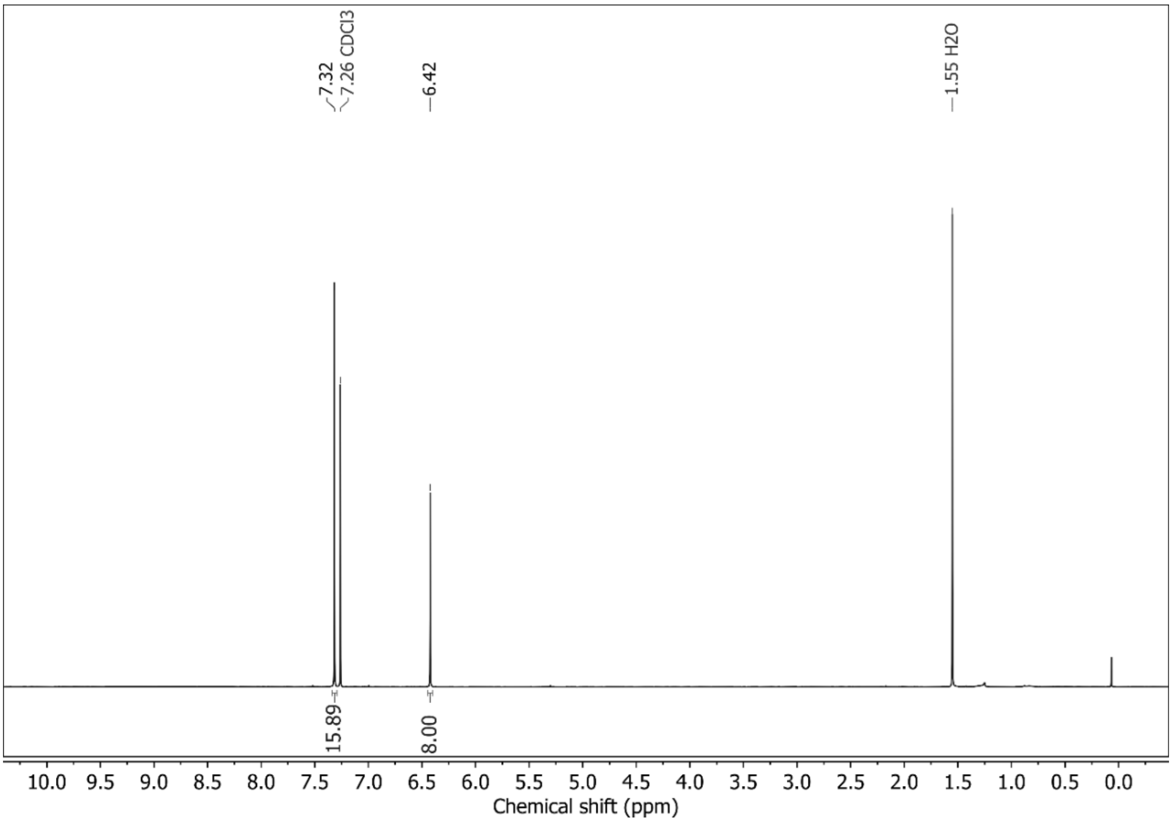

Figure S2. ^{1}H NMR (400 MHz, $CDCl_3$) of **PCT** purified by sublimation.

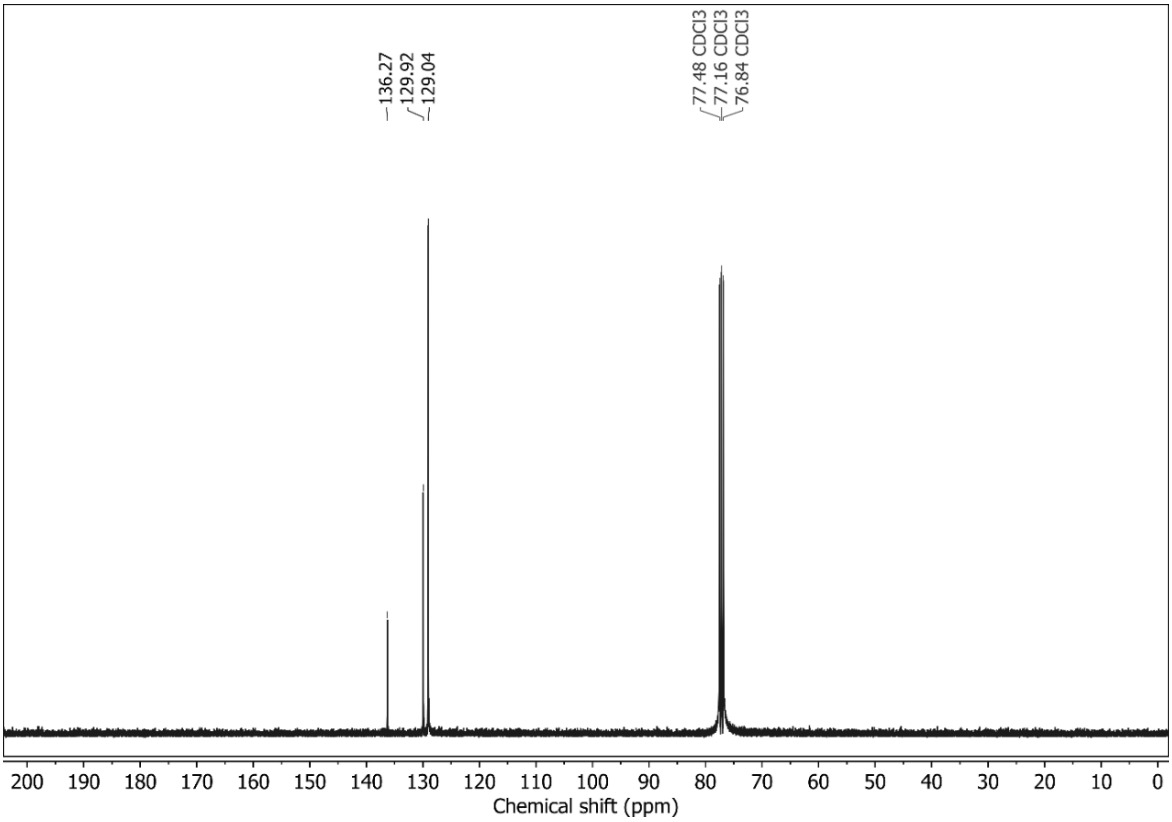

Figure S3. ^{13}C NMR (100 MHz, $CDCl_3$) of **PCT** (purified by GPC).

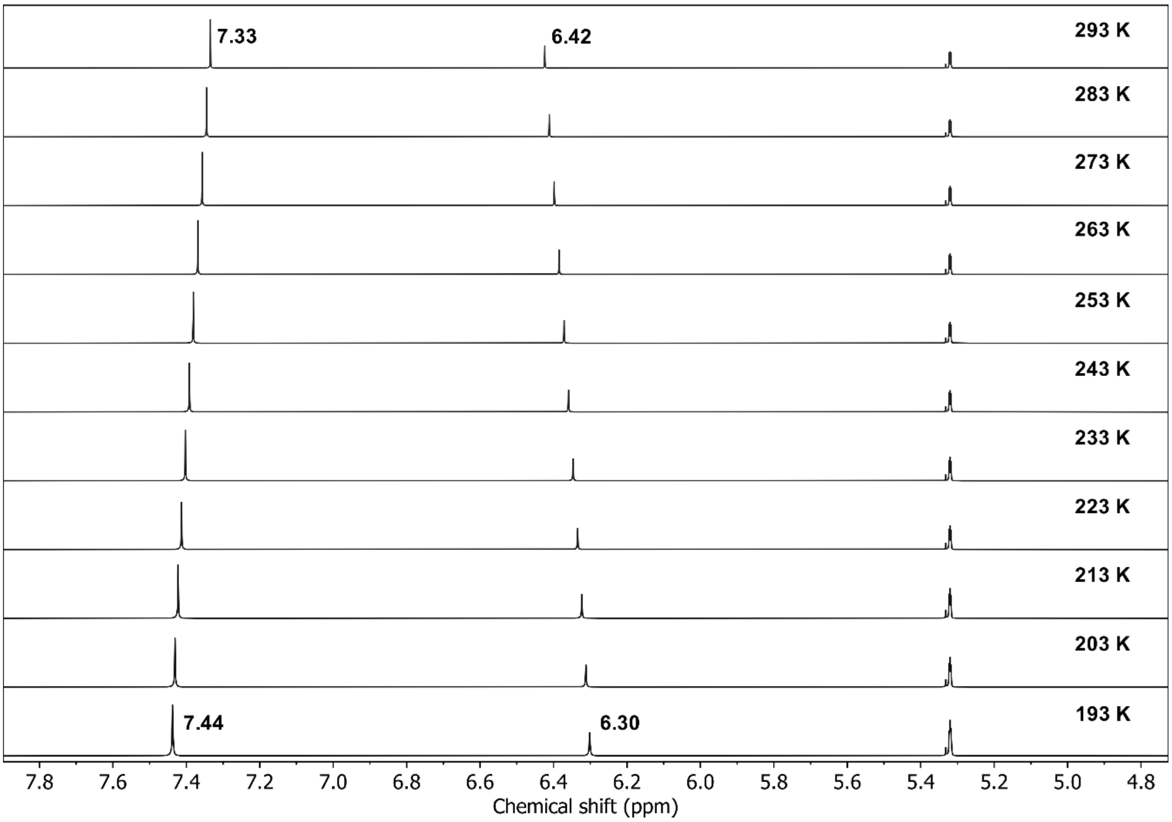

Figure S4. ^{1}H NMR (400 MHz, CD_2Cl_2) of **PCT** (purified by GPC) at temperatures from 293 K to 193 K. CD_2Cl_2 was used as the solvent for these measurements due to its lower melting point.

3 Thermal analysis

Thermogravimetric analysis (TGA) was carried out on a Mettler Toledo TGA/DSC 1LF/UMX at a heating rate of 10 °C min^{-1} under a nitrogen flow of 50 mL min^{-1}.

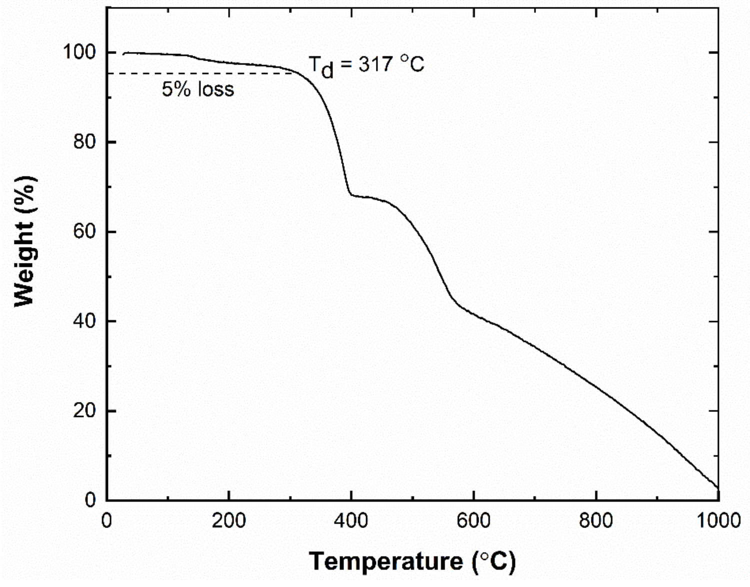

Figure S5. Thermogravimetric analysis (TGA) of **PCT**.

Differential scanning calorimetry (DSC) measurements were carried out on a TA DSC-Q20 instrument in a temperature range of 40 °C to 280 °C under a nitrogen flow of 50 mL min^{-1}. A conventional Tzero aluminum pan was used and heat flow measured at a heating rate of 10 °C min^{-1}.

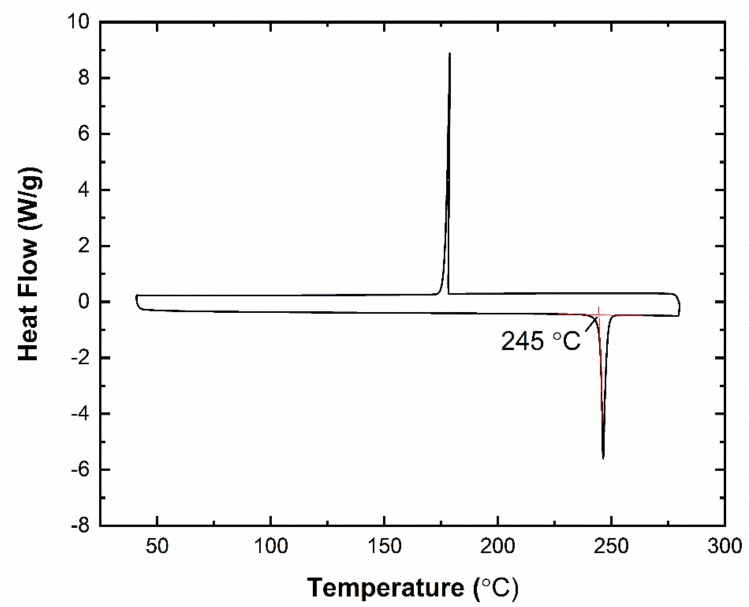

Figure S6. Second cycle of the differential scanning calorimetry (DSC) measurement of **PCT**.

4 Absorption and photoluminescence spectroscopy

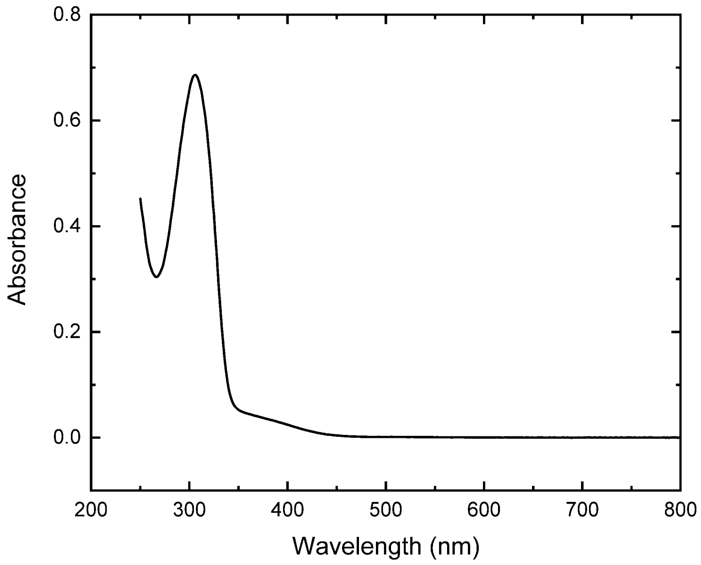

Figure S7. UV-vis absorption spectrum of **PCT** dissolved in $CHCl_3$ (λ_{max} = 306 nm).

The photoluminescence of **PCT** (λ_{ex} = 305 nm) was found to be very weak in $CHCl_3$ and no spectrum could be recorded.

5 Computational analysis

Geometry optimizations for neutral **PCT** and the different anionic and cationic states (**PCT**$^{2-/+}$, **PCT**$^{4-/+}$, **PCT**$^{6-/+}$) were performed using Gaussian 16 with the hybrid correlation-exchange functional PBE0[S2] and Def2-TZVP basis sets, as developed by Ahlrichs and Weigend.[S3] In all cases, molecules were relaxed using the *very tight* keyword, in which root mean square forces are relaxed until a threshold of 10^{-6} Hartree/Bohr is achieved. To ensure a true local minimum on the corresponding potential energy surfaces, we have performed a frequency analysis, revealing no imaginary frequencies. Singlet and triplet excited states for all charged states were computed with time-dependent DFT (TD-DFT) calculations at the same level of theory. Anisotropy of the induced current density (ACID) calculations were then performed on the optimized structures. The current densities were computed using the Continuous Set of Gauge Transformations (CSGT) method, as implemented in Gaussian 16.[S2a]. The induced current vectors and the corresponding plots were obtained with the ACID 3.0.0 program.[S4] The external magnetic field was applied perpendicular to the molecular plane.

5.1 Geometry optimization

We found that the optimized molecular structures belong to the symmetry point group D_{2d}. However, we can report distortions from the original neutral structure in the different reduced and oxidized states such as changes in dihedral angles. This can be observed in Figure S8, where we determined the root mean square displacement (RMSD) of the oxidized/reduced structures compared to the neutral structure.

To ensure the correct electronic ground state symmetry, we relaxed and computed the total energy for the triplet states of all cases employing restricted and unrestricted DFT. In these calculations, we have consistently found a singlet symmetry state as the electronic ground state in all cases. The results of these calculations are displayed in Table S1. We also analyzed structural changes due to different spin states (Figure S8).

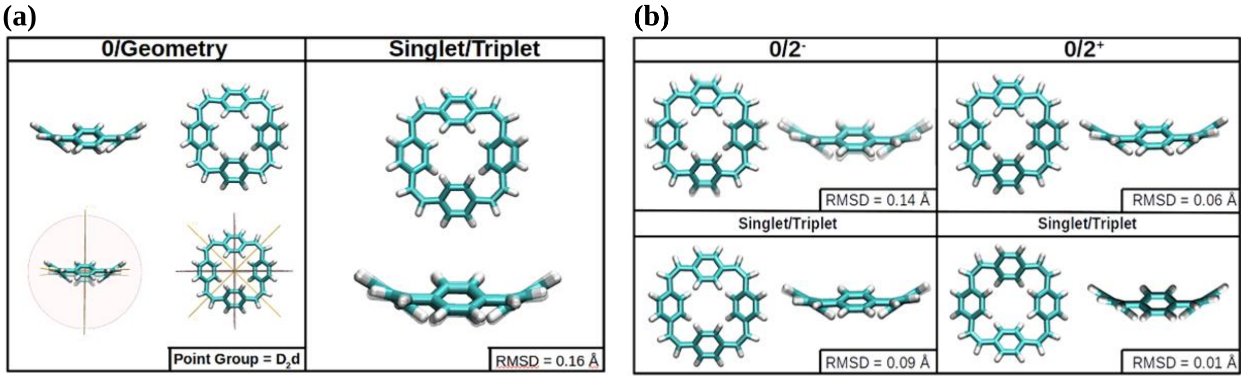

(c) (d)

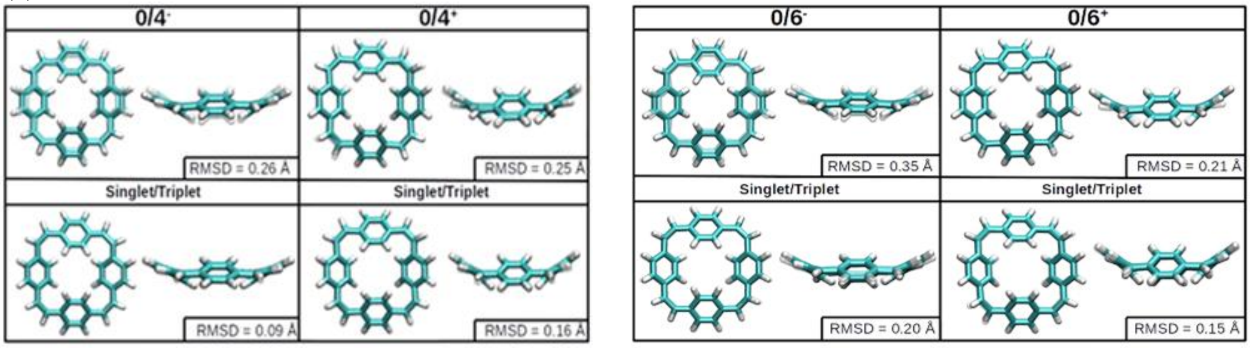

Figure S8. (a) Optimized geometry of neutral **PCT** obtained from DFT calculations indicating the symmetry point group. Comparison of the optimized geometries of (b) **PCT**$^{2-/+}$ and **PCT**, (c) **PCT**$^{4-/+}$ and **PCT**, and (d) **PCT**$^{6-/+}$ and **PCT** and comparison of the optimized geometries of the singlet and triplet states.

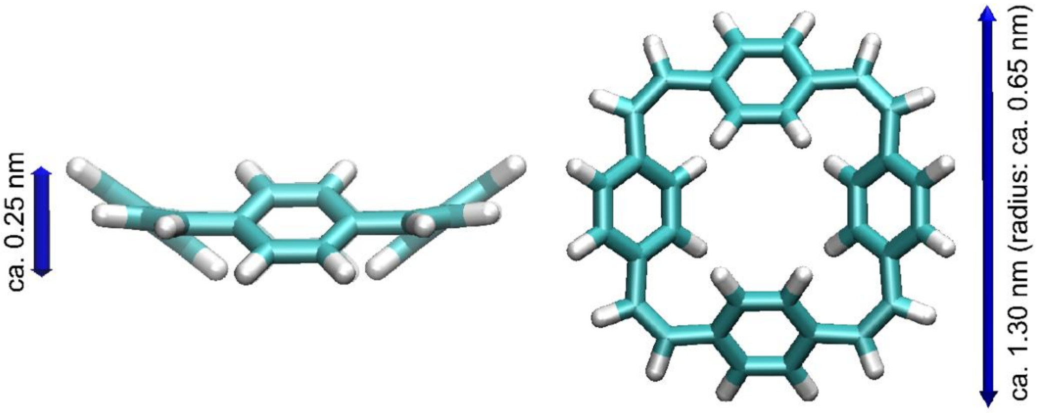

Figure S9. Optimized geometry of neutral **PCT** with scalebars showing the height (ca. 0.25 nm) and the diameter (ca. 1.30 nm, radius: ca. 0.65 nm) of the molecule.

Table S1. Total energies reported from optimized structures at the PBE0/Def2-TZVP level of theory.

Electronic state	$E_{triplet}$ (a.u.)	$E_{singlet}$ (a.u.)	$\Delta E_{triplet-singlet}$ (a.u.)
**0**	-1231.45442	-1231.52005	0.065
**2^-**	-1231.48625	-1231.51223	0.025
**2^+**	-1230.91581	-1230.93571	0.019
**4^-**	-1231.01856	-1231.02315	0.004
**4^+**	-1230.73330	-1230.74205	0.008
**6^-**	-1230.07176	-1230.09826	0.026
**6^+**	-1229.18712	-1229.19398	0.006

5.2 Electronic structure

The computation of the electronic structure of **PCT** was carried out at the PBE0/Def2-TZVP level of theory and a schematic representation of the energy levels is displayed in Figure S10. The three highest

occupied molecular orbitals can be divided into one independent electronic state (HOMO) and two degenerate states (HOMO-1) separated by an energy gap of 0.8 eV. Based on the results of the natural population analysis employing the NBO approach,[S5] we can conclude that all carbon atoms within the macrocycle are involved in π-bonds.

The three lowest unoccupied molecular orbitals can also be divided into an independent state (LUMO) and two degenerate states (LUMO+1), mirroring the behavior observed for the occupied energy levels.

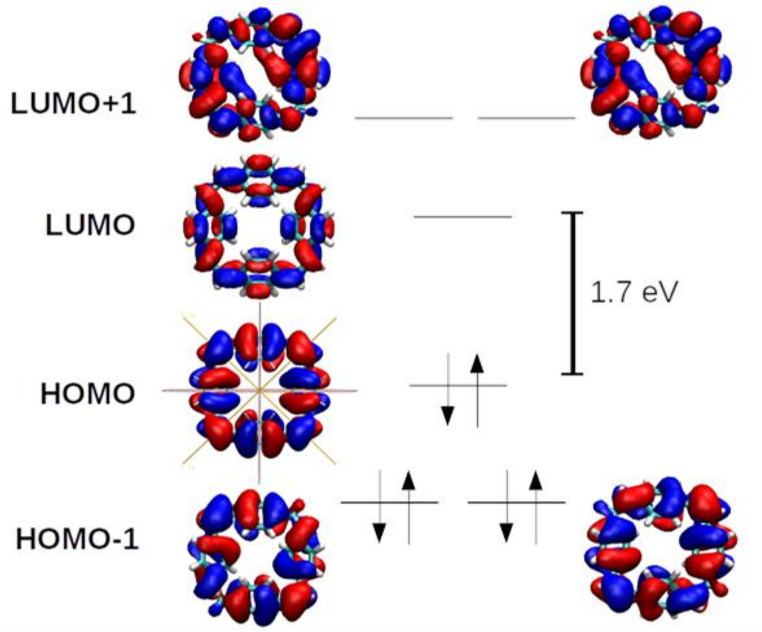

Figure S10. Electronic structure of neutral **PCT** for the three highest occupied molecular orbitals and three lowest unoccupied molecular orbitals.

5.3 Anisotropy of the induced current density (ACID) plots

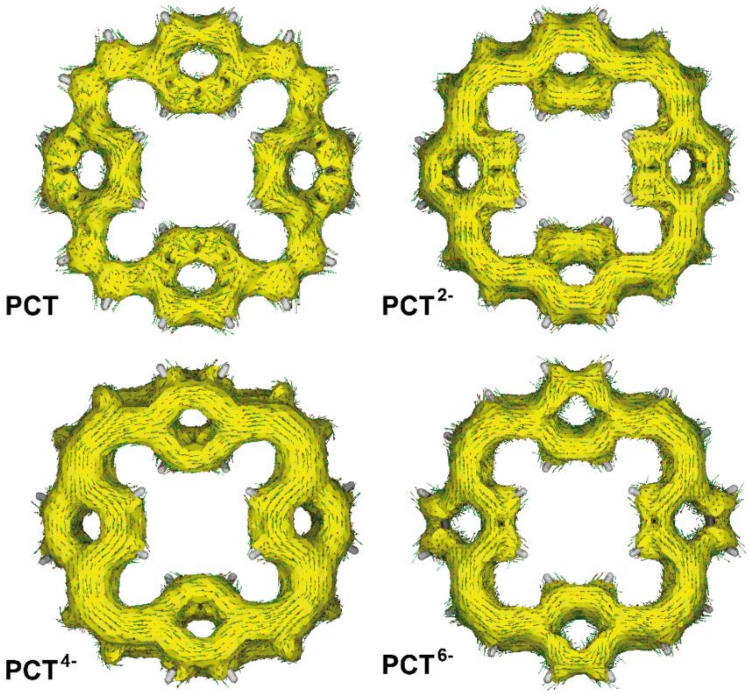

Figure S11. ACID plots of neutral **PCT** and anions **PCT^{2-}**, **PCT^{4-}** and **PCT^{6-}** at an isovalue of 0.04. Depending on the state, current density vectors indicate diatropic (clockwise, aromatic) or paratropic (antiaromatic, counter-clockwise) ring currents.

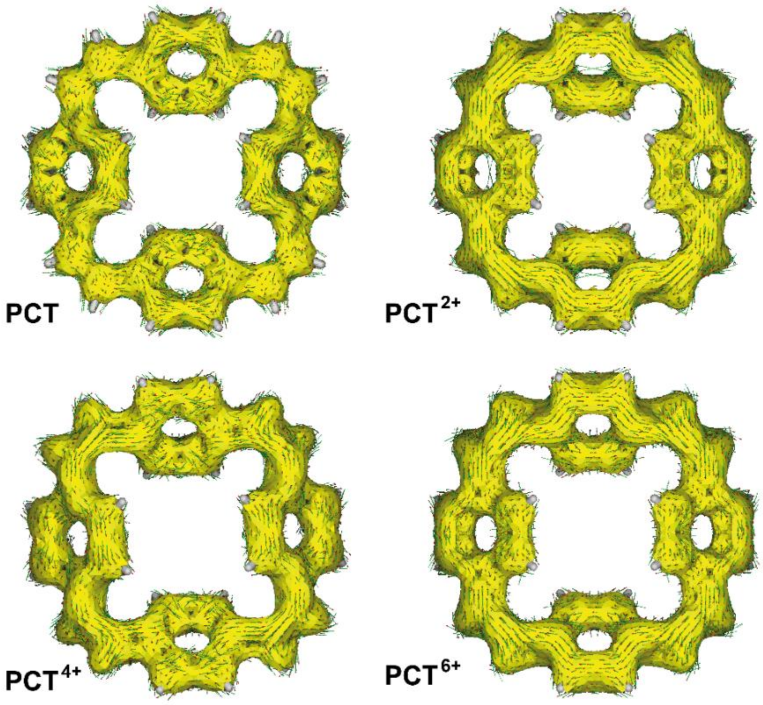

Figure S12. ACID plots of neutral **PCT** and cations **PCT^{2+}**, **PCT^{4+}** and **PCT^{6+}** at an isovalue of 0.04. Depending on the state, current density vectors indicate diatropic (clockwise, aromatic) or paratropic (antiaromatic, counter-clockwise) ring currents.

6 Cyclic voltammetry (CV) in solution

All voltammetric measurements (except for the measurement in Figure S13, details of this measurement are provided in the Supporting Information) were performed in an argon atmosphere glovebox (LabStar, MBraun) using a PalmSens4 potentiostat controlled via Bluetooth connection in a standard three electrode setup. Platinum disc ultramicroelectrodes (UMEs) were prepared by sealing 25 μm or 100 μm diameter microwires into borosilicate capillaries (Sutter Instruments, o.d. 1.2 mm, i.d 0.69 mm) under vacuum using a micropipette puller (PC-10, Narishige). The obtained composite was cut with a diamond knife and finally polished with 0.1 μm grade diamond lapping tape (Buehler) using a homemade microelectrode polisher.

Pt UME, Ag wire and Pt wire served as working, quasi-reference and auxiliary electrodes, respectively. Small glass test tube vessels were used as open electrochemical cells. The electrolyte volume was below 0.5 mL. After the measurements, an arbitrary amount of ferrocene (internal reference) was added to the solution in order to evaluate the redox potentials of the studied compound.

Cyclic voltammograms for kinetic parameters evaluation were recorded using a 2 mm diameter platinum disc electrode and fitted to simulated voltammograms using DigiSim 3.03b Software (Bioanalytical Systems). Ag wire and Pt wire again served as quasi-reference and auxiliary electrode, respectively, and ferrocene was added to evaluate the redox potentials.

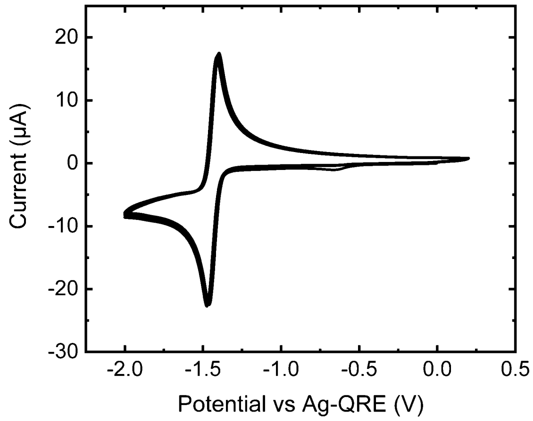

Figure S13. Cyclic voltammogram of **PCT** (first 30 cycles) recorded with a glassy carbon working electrode, platinum mesh auxiliary electrode, and Ag wire quasi-reference electrode (QRE). Supporting electrolyte: 0.1 M NBu_4PF_6 in DMF. Scan rate: 0.1 V s^{-1}. The solution was purged with nitrogen prior to the measurement for 5 min.

6.1 Electroreduction on platinum disc ultramicroelectrodes (UMEs)

PCT solutions of known concentrations (Table S2, *c*) in 0.1 M NBu_4PF_6 in *N*,*N*-dimethylformamide (DMF), propylene carbonate (PC) and 1,2-dichloroethane (DCE) were prepared for CV using platinum

disc ultramicroelectrodes (UMEs) of 25 μm diameter. The diffusion limited current (in A) at disc UMEs is given by Equation 1,[S6]

$$i_{ss} = 4nFDcr \quad \text{(Equation 1)}$$

where n is the number of electrons transferred per analyte molecule, F is the Faraday constant (96485 C mol^{-1}), D is the diffusion coefficient of the analyte (in cm^2 s^{-1}), c is the concentration of the analyte (in mol cm^{-3}) and r is the radius of the UME (in cm). We estimated the diffusion coefficient of **PCT** assuming a two-electron stoichiometry (n = 2) of the electroreduction of **PCT** (Table S2, D). Using literature data for the dynamic viscosities of the solvents, the obtained diffusion coefficients and the Stokes-Einstein equation we estimated the hydrodynamic radii of **PCT** in aforementioned solutions. The values, presented in Table S2, are in good agreement with the (non-spherical) geometry of **PCT** (Figure S9). The variation of the hydrodynamic radii in different solvents is considered to be a result of different intermolecular interaction between **PCT** and the solvent molecules. The larger hydrodynamic radii of **PCT** in DMF and PC compared to DCE may be caused by stronger attractive interaction of **PCT** with DMF/PC molecules (e.g. interactions of π-orbitals) than with DCE molecules.

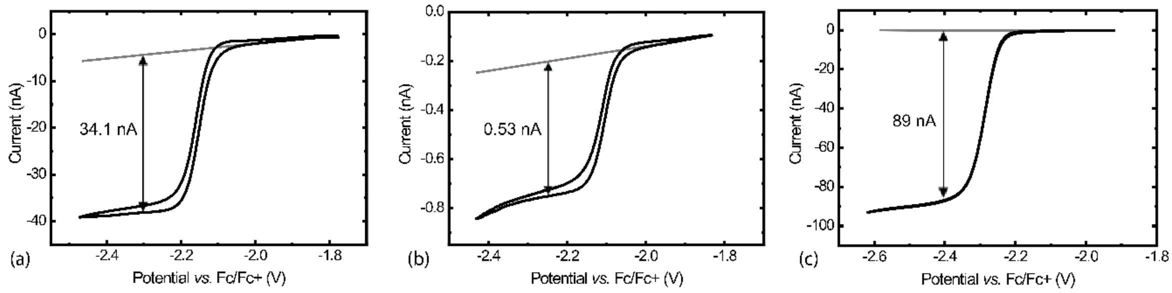

Figure S14. Cyclic voltammograms of **PCT** recorded on platinum disc UMEs of 25 μm diameter in (a) DMF, (b) PC and (c) DCE. Supporting electrolyte: 0.1 M NBu_4PF_6. **PCT** concentrations provided in Table S2. Scan rate: 10 mV s^{-1}. Potential shown versus the half-wave potential of ferrocene (added to the solutions after initial measurements).

Table S2. Physicochemical parameters of electroreduction of **PCT** (diffusion coefficient, D; concentration, c; steady state current at disc UME of 25 μm diameter, i_{SS}; hydrodynamic radius, r_H; formal potential, E^{0}'; number of electrons transferred per molecule, n; standard electron transfer rate constant, k_0; electron transfer coefficient, α) in three different solvents (dynamic viscosity, η; dipole moment, μ) at 298.15 K. Data related to CV using UMEs highlighted in light grey; kinetic parameters from fitting simulated voltammograms to voltammograms obtained using platinum disc electrodes of 2 mm diameter highlighted in dark grey.

Solvent	η (mPa·s)	μ (D)	D (cm^2 s^{-1})	c (mmol dm^{-3})	i_{ss} (nA)	r_H (nm)	n	k_0 (cm/s)	α	E^{0}'(**PCT**$^{2-}$/**PCT**) *vs* Fc/Fc$^+$ (V)
N,*N*-Dimethylformamide (DMF)	0.864 [S7]	3.82 [S8]	4.87 × 10^{-6}	7.25	34.1	0.51	2	1.8 × 10^{-3}	0.17	-2.155
Propylene carbonate (PC)	2.4711 [S9]	5.36 [S10]	1.70 × 10^{-6}	0.32	0.53	0.51	2	1.8 × 10^{-3}	0.32	-2.105
1,2-dichloroethane (DCE)	0.7644 [S11]	2.94 [S8]	7.20 × 10^{-6}	12.8	89	0.39	2	1.0 × 10^{-3}	0.29	-2.285

6.2 Electroreduction on platinum disc electrodes of 2 mm diameter

Kinetic parameters were determined by fitting simulated voltammograms to cyclic voltammograms recorded using 2 mm diameter platinum disc electrodes. As **PCT**$^{2-}$ can be oxidized back to its neutral form **PCT** by air/oxygen remaining in the solvent,[S12] we used the EC' mechanism for fitting. This mechanism includes the homogeneous regeneration of **PCT** shown in Equation 3. The concentrations (same as for the measurements using UMEs) and diffusion coefficients (determined from the measurements using UMEs) were fixed (not fitted).

$$\mathbf{PCT} + 2e^- \overset{k_0}{\rightleftharpoons} \mathbf{PCT}^{2-} \qquad \text{(Equation 2)}$$

$$\mathbf{PCT}^{2-} \text{ (+ oxidant)} \overset{k}{\rightarrow} \mathbf{PCT} \text{ (+ reduced oxidant)} \qquad \text{(Equation 3)}$$

As the concentration of remaining oxygen or other oxidants is unknown, we used a pseudo first order homogeneous conversion of **PCT**$^{2-}$ to **PCT** (Equation 3). The best fits were obtained with a rate constants k of 0.046 s^{-1} (in DMF), 0.721 s^{-1} (in PC) and 0.237 s^{-1} (in DCE), reflecting rather slow kinetics but influencing the shape of the voltammograms and the fitting of kinetic parameters. The standard electron transfer rate constants k_0 and electron transfer coefficients α are listed in Table S2.

6.3 Determination of formal potentials

The formal potentials of the **PCT**$^{2-}$/**PCT** redox couple in the different solvents (Table S2, $E^{0\prime}$) were determined using ferrocene (Fc) as the reference. After recording cyclic voltammograms with a silver quasi-reference electrode (QRE), an arbitrary amount of ferrocene was added to the electrolyte and another voltammogram was recorded. In order to find the formal potentials, we differentiated the cyclic voltammograms to determine their peak potentials precisely (see Figure S15 for example).

There is a correlation between measured formal potentials of the **PCT**$^{2-}$/**PCT** redox couple and the dipole moments of the solvents. Different Gibbs free energies of the solvation of **PCT** and **PCT**$^{2-}$ (as well as of ferrocene and ferrocene$^+$) in different solvents affect the thermodynamics of the studied reactions and result in different redox potentials in different solvents.

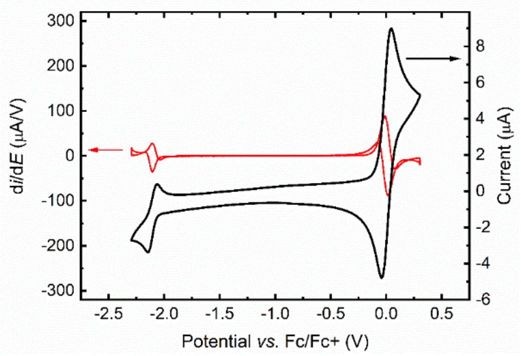

Figure S15. Cyclic voltammogram recorded in PC on a 2 mm diameter Pt disc electrode and its 1st derivative. Scan rate: 0.1 V s^{-1}. Supporting electrolyte: 0.1 M NBu_4PF_6.

6.4 Electrooxidation on platinum disc ultramicroelectrodes (UMEs)

The electrooxidation of **PCT** (Equation 4) is chemically irreversible in DMF and PC (Figure S16a and S16b). The anodic current of the oxidation of **PCT** is very similar to the cathodic current of the reduction in both solvents, indicating that the oxidation of **PCT** is also a two-electron process. The formal potential of the **PCT**/**PCT**$^{2+}$ redox couple is estimated as E^{0}'(**PCT**/**PCT**$^{2+}$) ≈ 0.75 V vs Fc/Fc^{+} (in DMF) and 0.77 V vs Fc/Fc^{+} (in PC) from the inflection points. In both solvents, an electroactive compound with sluggish electron transfer kinetics around -1 V vs Fc/Fc^{+} remains on the electrode after the first oxidation, which causes a decrease of the **PCT** reduction current in the second scan (Figure S16b, dotted line).

$$\mathbf{PCT} \rightleftharpoons \mathbf{PCT}^{2+} + 2e^- \qquad \text{(Equation 4)}$$

In DCE, the electrooxidation of **PCT** is chemically reversible, but oxidized **PCT** adsorbs to the electrode, causing substantial deceleration of the charge transfer rate (Figure S16c). However, as in the other solvents, the anodic peak current is very similar to the cathodic current, confirming the two-electron nature of the oxidation. The formal potential of the **PCT**/**PCT**$^{2+}$ redox couple is estimated as E^{0}'(**PCT**/**PCT**$^{2+}$, DCE) ≈ 0.77 V vs Fc/Fc^{+} from the inflection point.

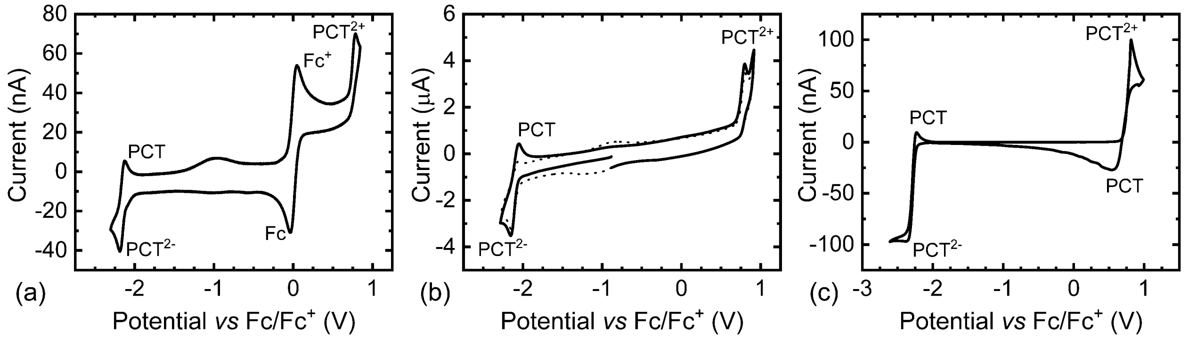

Figure S16. Cyclic voltammograms of **PCT** recorded (a) in DMF on a 25 µm UME (with ferrocene as internal reference, scan rate: 1 V s^{-1}), (b) in PC on a 2 mm disc electrode (scan rate: 0.1 V s^{-1}) and (c) in DCE on a 25 µm UME (7.25 mM **PCT**, scan rate: 0.1 V s^{-1}). Supporting electrolyte: 0.1 M NBu_4PF_6.

7 Crystallography

Distances between least-square planes defined by the atoms of **PCT** molecules as well as voids in the crystal structures were calculated and displayed using the respective tools in Mercury CSD 4.0.0.

7.1 PCT single crystal grown from acetic acid solution

Single crystals grown by slowly cooling down a saturated boiling solution of **PCT** in acetic acid. *Crystal data*: $C_{32}H_{24}$, M = 408.51, monoclinic, $P2_1$ (no. 4), a = 11.7806(12), b = 6.2488(7), c = 16.6280(15) Å,

β = 109.573(10)°, V = 1153.3(2) Å^3, Z = 2, D_c = 1.176 g cm^{-3}, μ(Mo-Kα) = 0.066 mm^{-1}, T = 173 K, yellow tabular needles, Agilent Xcalibur 3 E diffractometer; 3387 independent measured reflections (R_{int} = 0.0194), F^2 refinement,[S13] R_1(obs) = 0.0866, wR_2(all) = 0.2051, 1963 independent observed absorption-corrected reflections [$|F_o| > 4\sigma(|F_o|)$, completeness to θ_{full}(67.7°) = 98.9%], 328 parameters. The absolute structure could not be determined [Flack parameter x = –8.0(10)]. CCDC 1985103.

The whole of the structure was found to be disordered. Two orientations were identified of *ca*. 56 and 44% occupancy (Figure S17), the geometries of the aryl rings of both orientations were idealized, the thermal parameters of adjacent atoms were restrained to be similar, and only the non-hydrogen atoms of the major occupancy orientation were refined anisotropically (those of the minor occupancy orientation were refined isotropically). A weak restraint was applied to all of the anisotropic atoms to restrain their thermal parameters to be approximately isotropic.

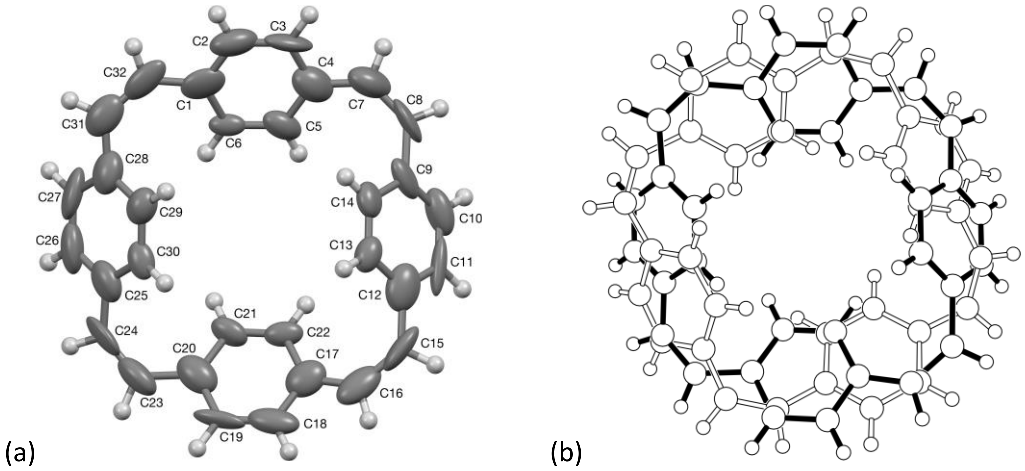

Figure S17. (a) Crystal structure of **PCT** single crystal grown from acetic acid solution (50% probability ellipsoids). (b) Overlay of the two partial occupancy orientations present in the crystal structure of **PCT** (the *ca*. 56% occupancy major orientation is drawn with dark bonds, the *ca*. 44% occupancy minor orientations is drawn with open bonds).

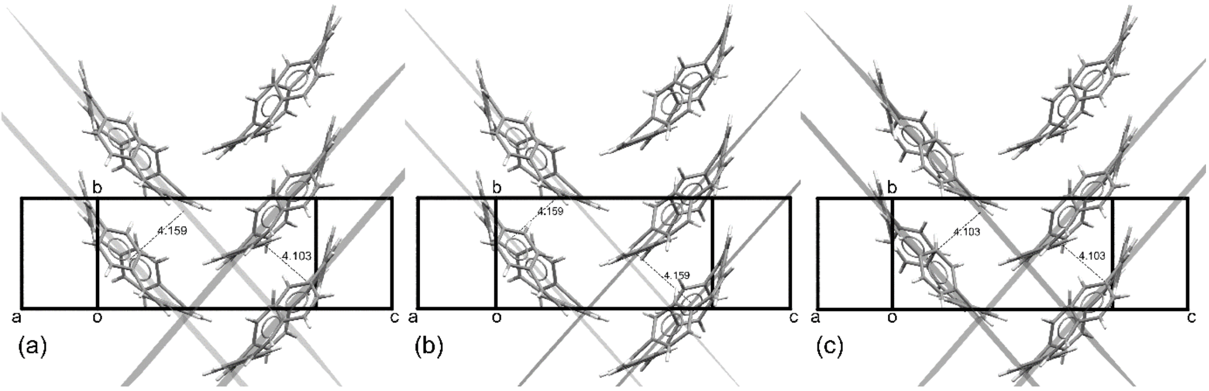

Figure S18. Crystal structure of **PCT** single crystal grown from acetic acid solution showing distances between **PCT** molecules when (a) one of the two **PCT** molecules of the unit cell adopts orientation A and the other one orientation B (4.159 Å and 4.103 Å), (b) both molecules adopt orientation A (4.159 Å), (c) both molecules adopt orientation B (4.103 Å).

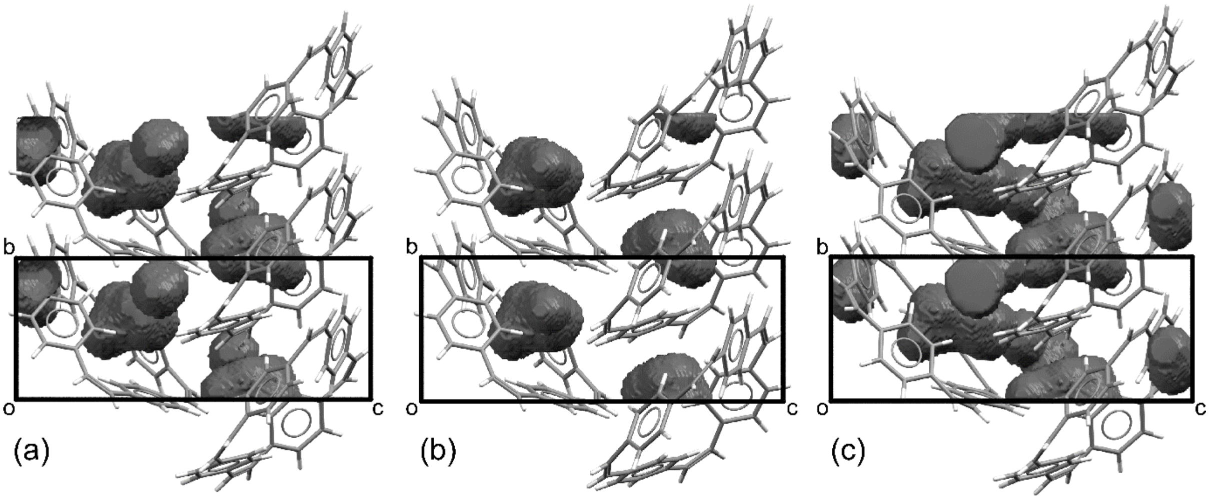

Figure S19. Crystal structure of **PCT** single crystal grown from acetic acid solution (viewed along the *a*-axis) showing voids large enough to contain Na^+ ions when (a) one of the two **PCT** molecules of the unit cell adopts orientation A and the other one orientation B (5.8 % of unit cell volume), (b) both molecules adopt orientation A (4.1 % of unit cell volume), (c) both molecules adopt orientation B (8.1 % of unit cell volume).

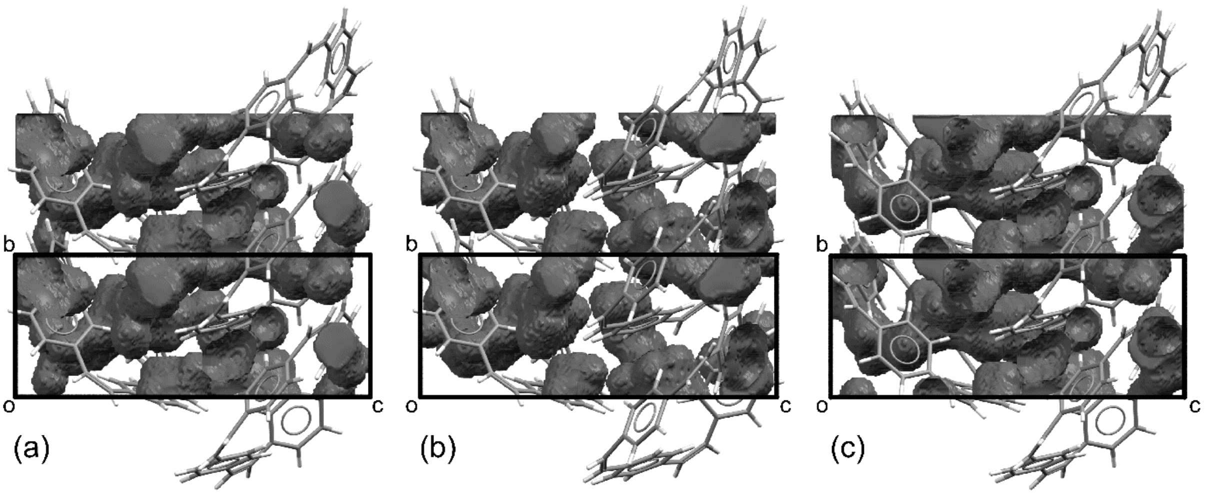

Figure S20. Crystal structure of **PCT** single crystal grown from acetic acid solution (viewed along the *a*-axis) showing voids large enough to contain Li^+ ions when (a) one of the two **PCT** molecules of the unit cell adopts orientation A and the other one orientation B (15.5 % of unit cell volume), (b) both molecules adopt orientation A (14.1 % of unit cell volume), (c) both molecules adopt orientation B (17.0 % of unit cell volume).

7.2 Previously reported PCT crystal structure

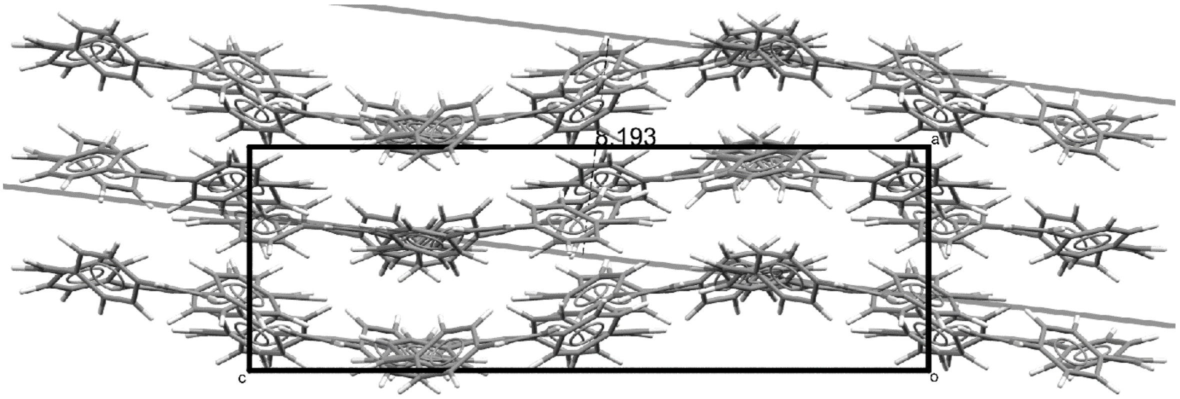

Figure S21. Previously reported crystal structure of **PCT** (viewed along the *b*-axis) showing the distance between two identical **PCT** molecules (8.193 Å) with one **PCT** molecule in between.

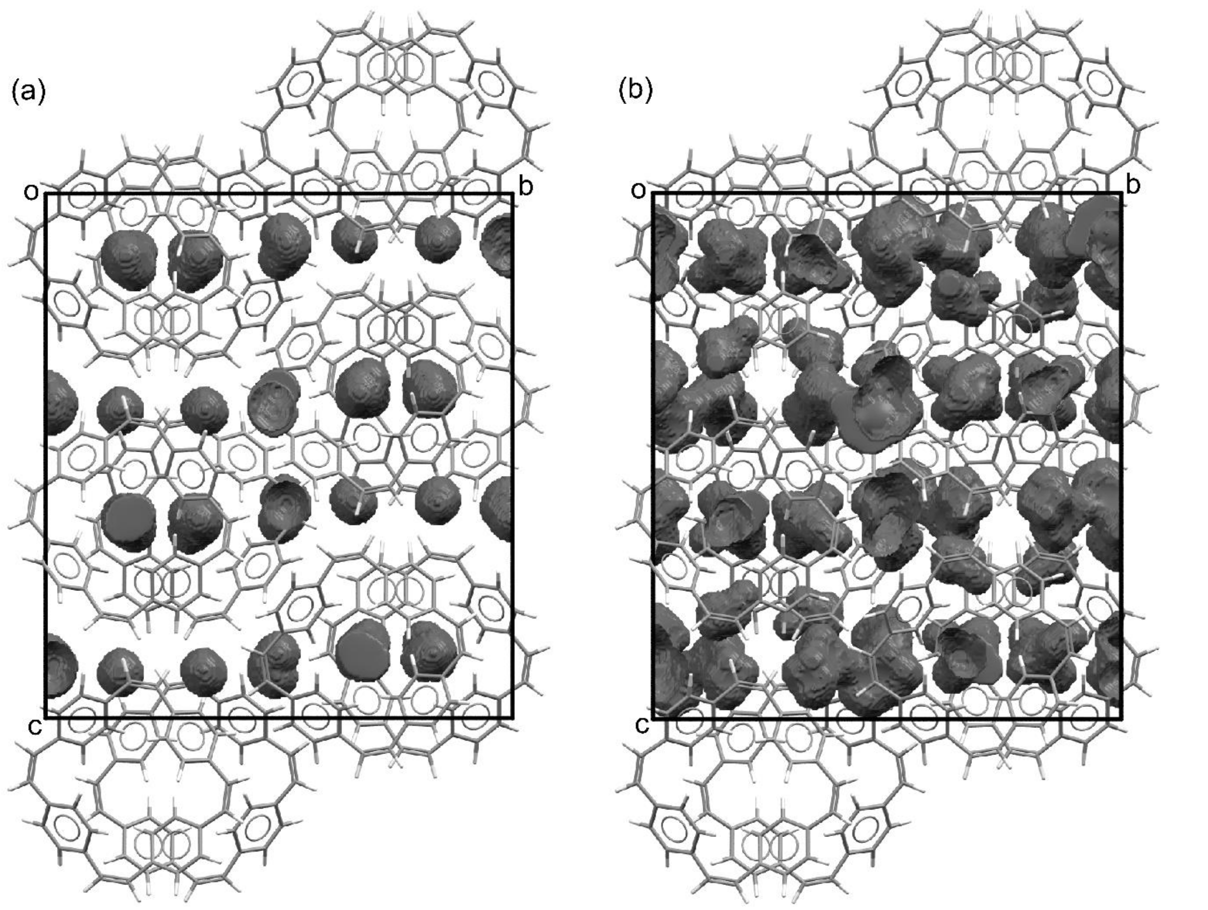

Figure S22. Previously reported crystal structure of **PCT** (viewed along the *a*-axis) showing voids large enough to contain (a) Na^+ ions (4.9 % of unit cell volume) and (b) Li^+ ions (14.9 % of unit cell volume).

7.3 PCT single crystal grown by sublimation

Single crystals grown by sublimation at approx. 0.40 mbar and 240°C. *Crystal data*: $C_{32}H_{24}$, M = 408.51, triclinic, $P1$ (no. 1), a = 12.3959(5), b = 13.2954(7), c = 31.4947(16) Å, α = 89.079(4), β = 89.893(4), γ = 62.415(4)°, V = 4599.9(4) Å^3, Z = 8 [8 independent molecules], D_c = 1.180 g cm^{-3}, μ(Cu-Kα) = 0.503 mm^{-1}, T = 173 K, yellow needles, Agilent Xcalibur PX Ultra A diffractometer; 13816 independent measured reflections (R_{int} = 0.0374), F^2 refinement,[S13] R_1(obs) = 0.0800, wR_2(all) = 0.2455, 12176 independent observed absorption-corrected reflections [$|F_o| > 4\sigma(|F_o|)$, completeness to θ_{full}(67.7°) = 67.7%], 2307 parameters. The absolute structure could not be determined [Flack parameter x = –3(3)]. CCDC 1985326.

The initial scans of this crystal strongly suggested that the correct Bravais lattice was *C*-face centered monoclinic [a = 23.571(3), b = 12.4006(12), c = 31.511(4) Å, α = 89.848(9), β = 90.948(9), γ = 89.821(8)°, V = 9209(2) Å^3, *mC*, 89% indexed (1889 out of 2126 observed spots used)]. A 15 hour experiment based on this unit cell gave a *ca*. 98.7% complete data set with an R_{int} of 0.090. (This compares to R_{int} values of > 0.40 for the related *oC*, transformed *mC*, and half-volume *mC* unit cells,

though the half-volume *aP* cell gave an R_{int} of 0.039.) Despite numerous efforts, however, no solution for this structure could be found using this *mC* unit cell.

In an effort to resolve the problem (and suspecting twinning), a new crystal was tried, but initial scans gave a different, poorly indexed, unit cell [a = 23.52(4), b = 12.40(2), c = 33.81(6) Å, α = 90.26(15), β = 111.35(16), γ = 89.98(15)°, V = 9181(20) $Å^3$, *mC*, 63% indexed (19 out of 30 observed spots used)]. (The data for the first crystal can be indexed to this new unit cell, but to only 48% completeness.) So, no data collection was run.

Later re-analysis of the data set from the first crystal finally gave a solution by dropping to the lowest symmetry (using the reduced triclinic cell and space group *P*1), though as a consequence of this lower symmetry the data set is only *ca*. 68% complete. The solved structure has eight independent molecules (**A** to **H,** Figure S23), all of which show distorted thermal ellipsoids suggesting similar disorder of each ring to that seen in the crystal structure of the **PCT** single crystal grown from acetic acid solution, though to a lesser extent. Here no serious effort to model this disorder was attempted, with only a few restraints used to "manage" the worst of the thermal ellipsoids. Whilst we are not fully convinced that this is the "correct" combination of unit cell and space group, it is the only combination that has led to any solution, and does allow for a reasonable refinement, and analysis of the coordinates has not revealed any higher symmetry. As such, despite the uncertainties and the incomplete data, we feel that the model presented gives reliable information about the relative positions of the eight macrocycles, allowing the packing to be sensibly analyzed.

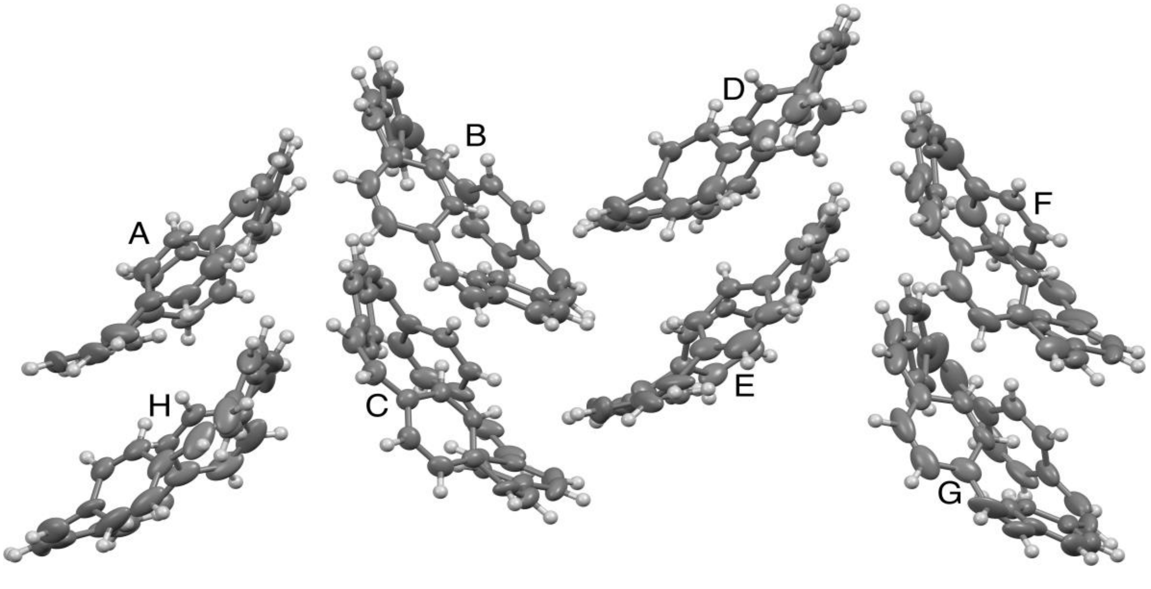

Figure S23. Crystal structure of the **PCT** single crystal grown by sublimation showing the eight independent molecules (**A** to **H**) in the asymmetric unit (50% probability ellipsoids).

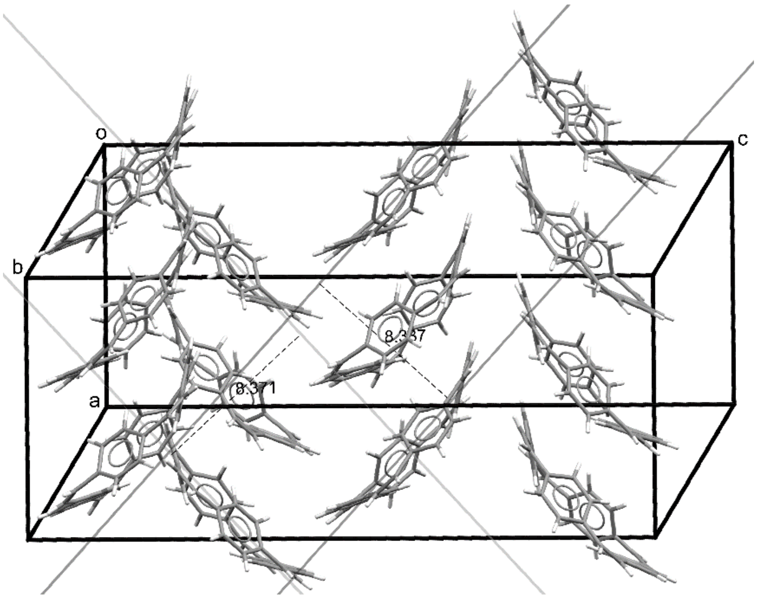

Figure S24. Crystal structure of the **PCT** single crystal grown by sublimation showing distances between identical **PCT** molecules (8.371 Å and 8.337 Å) with one **PCT** molecule in between.

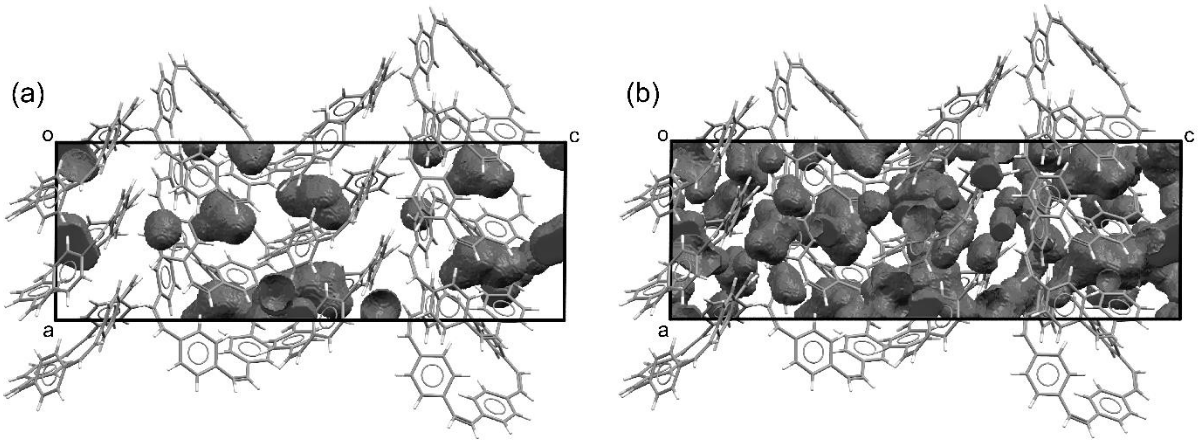

Figure S25. Crystal structure of the **PCT** single crystal grown by sublimation (viewed along the *b*-axis) showing voids large enough to contain (a) Na^+ ions (5.5 % of unit cell volume) and (b) Li^+ ions (14.9 % of unit cell volume).

7.4 Powder X-ray diffraction

Powder XRD data was collected on a Bruker D2 Phaser producing Cu-K_α (λ = 1.5406 Å) radiation, fitted with a LYNXEYE XE-T detector.

Measurement 1: Powder XRD pattern of **PCT** powder as obtained after purification by GPC. The pattern matched well with the pattern calculated from the single-crystal data of **PCT** reported previously (Figure S26a).

Measurement 2: Powder XRD pattern of crystals grown by slowly cooling down a boiling saturated solution of **PCT** in acetic acid ground into a fine powder in an agate mortar. The obtained pattern

matched well with the pattern calculated from the crystal data of our **PCT** single crystal grown from acetic acid (Figure S26b).

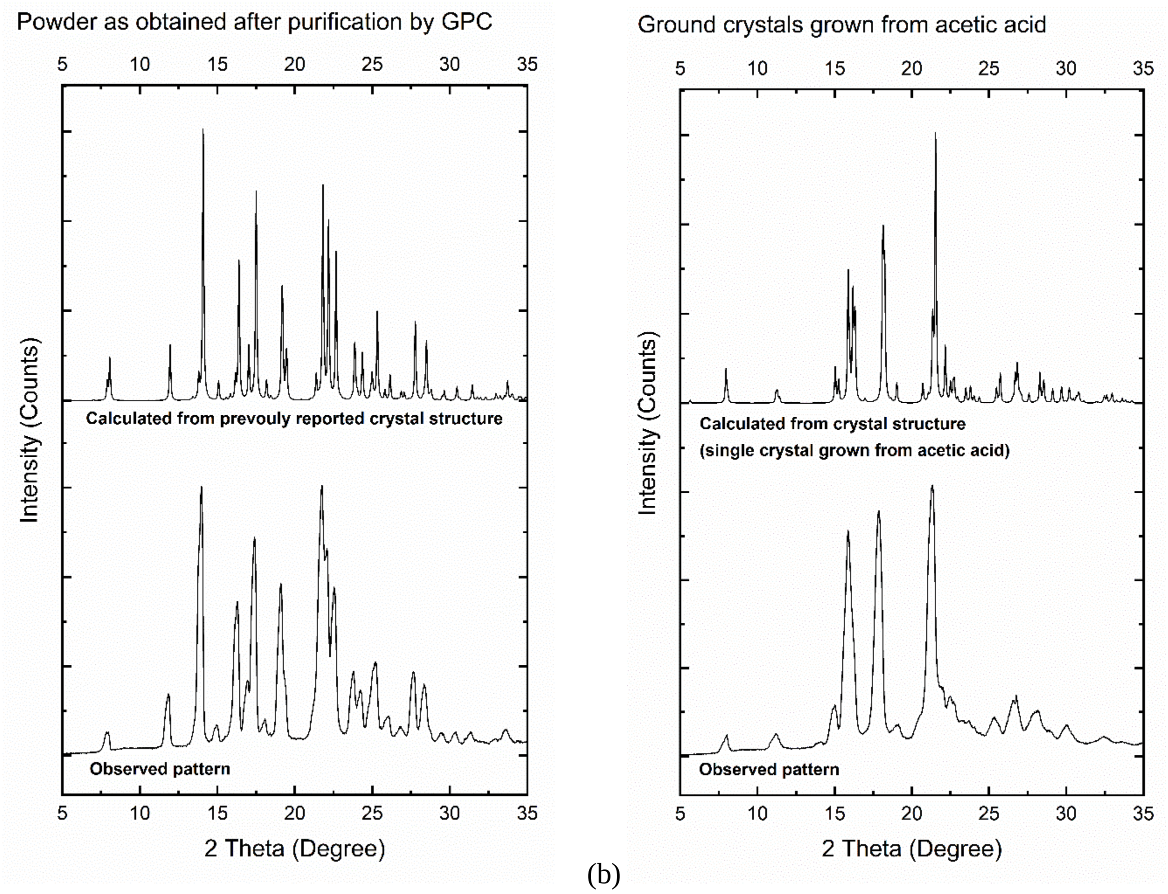

(a) (b)

Figure S26. Powder XRD patterns of (a) **PCT** powder as obtained after purification by GPC (Measurement 1) and (b) ground **PCT** crystals obtained from acetic acid solution (Measurement 2) compared to patterns calculated from single crystal data.

7.5 1,4-Distyrylbenzene crystal structure (for comparison)

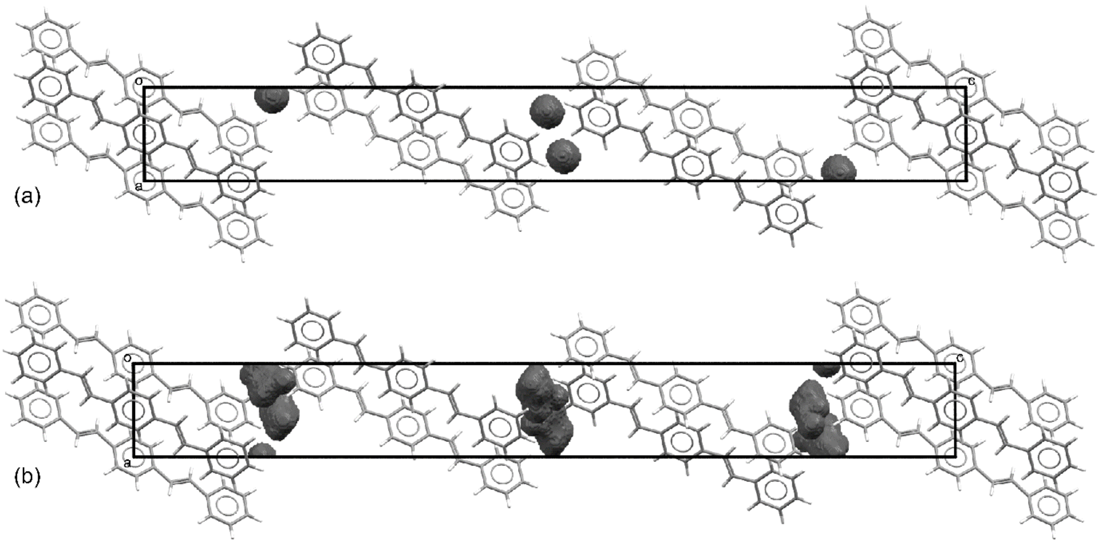

Figure S27. Crystal structure of 1,4-distyrylbenzene (viewed along the *b*-axis) showing voids large enough to contain (a) Na^+ ions (0.8 % of unit cell volume) and (b) Li^+ ions (3.1 % of unit cell volume).

8 Sodium-ion battery electrode tests and characterization

The sodium-ion battery (SIB) electrodes were prepared based on the following procedure: **PCT**, denka black, and poly(vinylidenedifluoride) (PVDF) were uniformly dispersed in *N*-methyl-2-pyrolidone (NMP) in a weight ratio of 3:5:2. The well-mixed slurry was cast onto Cu foil using the doctor blade method. The cast electrodes were then dried under vacuum at 80 °C for 12 h. The areal mass loading of the active material was 2.0 mg cm^{-2}. The electrochemical performance was characterized by fabricating 2032 coin-type cells with sodium metal discs paired as both counter and reference electrodes. The electrolyte used was 1 M sodium hexafluorophosphate ($NaPF_6$) in ethylene carbonate (EC) / diethyl carbonate (DEC) (1/1 = *v*/*v*) with 10 vol% fluoroethylenecarbonate (FEC). Cyclic voltammetry (CV) and galvanostatic measurements were performed using a battery cycler (WBCS3000L, Wonatech) at 25 °C. The chemical compositions of pristine, discharged and charged **PCT** electrodes were analyzed by XPS (sigma probe, Thermo VG scientific, England) with an Mg K α line as an X-ray source.

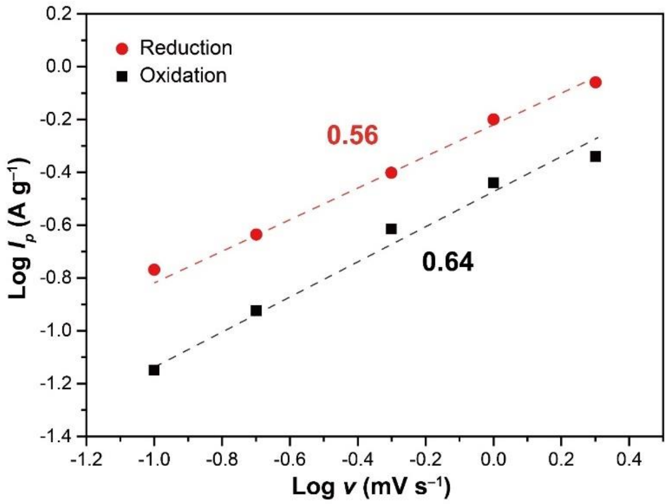

Figure S28. Log v-log I_p plots of data obtained from cyclic voltammetry at different scan rates to extract b-values based on the relation $I_p=av^b$ (v is the scan rate, I_p is the absolute value of the peak current density).

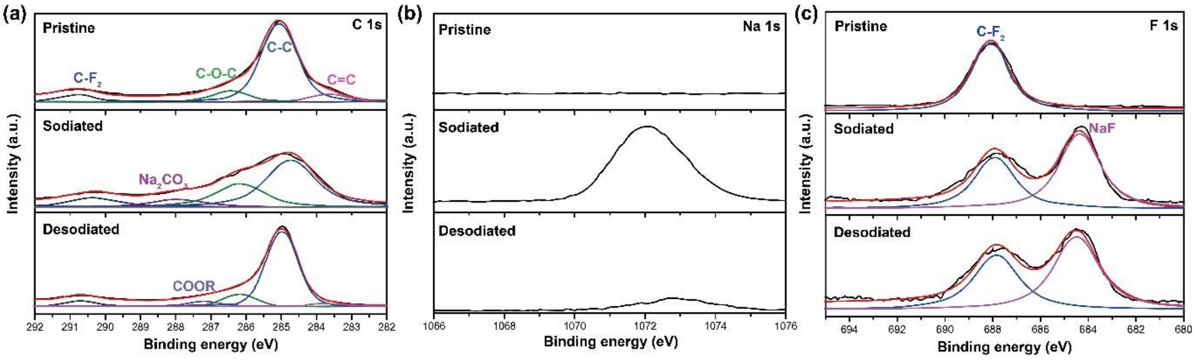

Figure S29. XPS spectra of pristine, sodiated, and desodiated **PCT** electrodes in (a) C 1s, (b) Na 1s, and (c) F 1s branches.

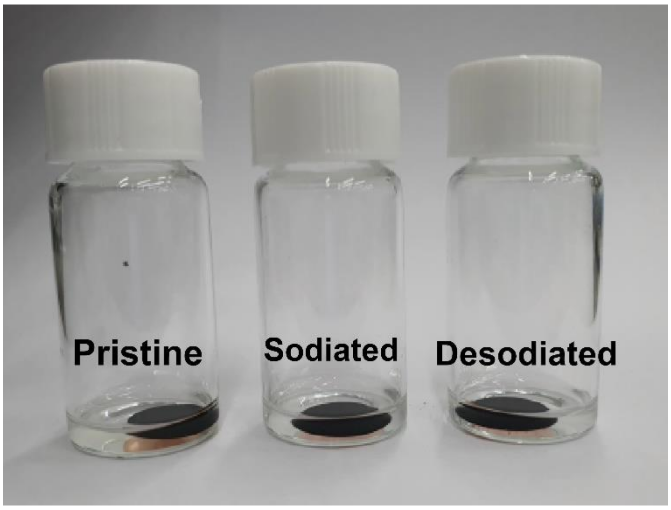

Figure S30. Photograph of pristine, sodiated, and desodiated **PCT** electrodes in the electrolyte.

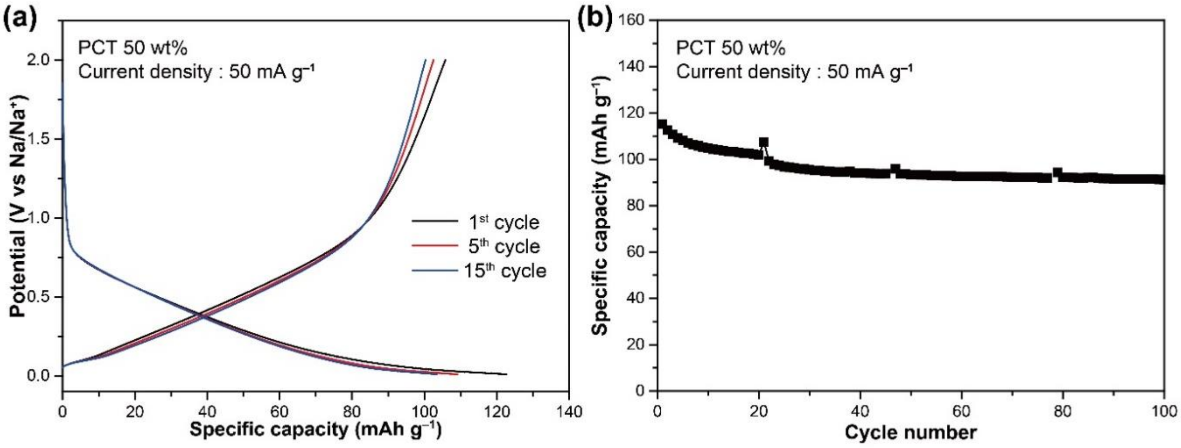

Figure S31. (a) Voltage profiles and (b) cycling performance of **PCT** electrode with 50 wt% of active material at a current density of 50 mA g^{-1}. The composition of the electrode is **PCT**:denka black:PVDF in a weight ratio of 5:4:1.

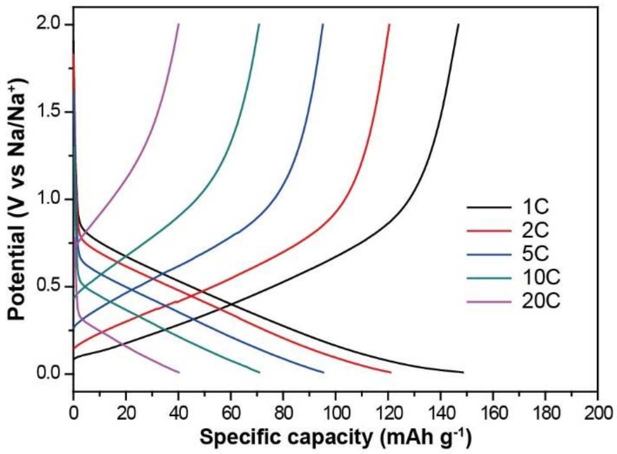

Figure S32. Voltage profiles of **PCT** when measured at various C-rates (1C = 100 mA g^{-1}).

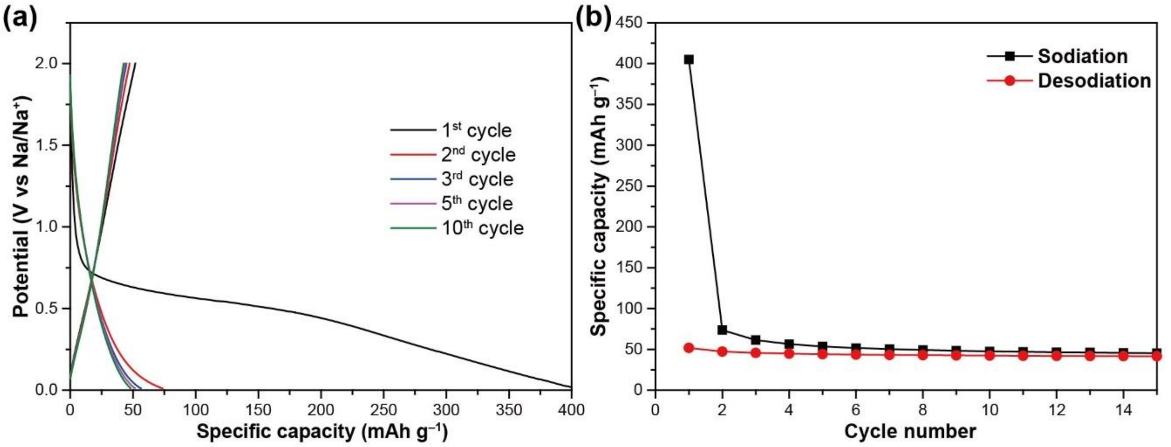

Figure S33. (a) Voltage profiles and (b) capacity retention of denka black at a current density of 200 mA g^{-1}.

9 References

[S1] B. Thulin, O. Wennerström, H.-E. Högberg, *Acta Chem. Scand., Ser. B* **1975**, *29*, 138-139. doi:10.3891/acta.chem.scand.29b-0138

[S2] a) M. J. Frisch, G. W. Trucks, H. B. Schlegel, G. E. Scuseria, M. A. Robb, J. R. Cheeseman, G. Scalmani, V. Barone, G. A. Petersson, H. Nakatsuji, X. Li, M. Caricato, A. V. Marenich, J. Bloino, B. G. Janesko, R. Gomperts, B. Mennucci, H. P. Hratchian, J. V. Ortiz, A. F. Izmaylov, J. L. Sonnenberg, Williams, F. Ding, F. Lipparini, F. Egidi, J. Goings, B. Peng, A. Petrone, T. Henderson, D. Ranasinghe, V. G. Zakrzewski, J. Gao, N. Rega, G. Zheng, W. Liang, M. Hada, M. Ehara, K. Toyota, R. Fukuda, J. Hasegawa, M. Ishida, T. Nakajima, Y. Honda, O. Kitao, H. Nakai, T. Vreven, K. Throssell, J. A. Montgomery Jr., J. E. Peralta, F. Ogliaro, M. J. Bearpark, J. J. Heyd, E. N. Brothers, K. N. Kudin, V. N. Staroverov, T. A. Keith, R. Kobayashi, J. Normand, K. Raghavachari, A. P. Rendell, J. C. Burant, S. S. Iyengar, J. Tomasi, M. Cossi, J. M. Millam, M. Klene, C. Adamo, R. Cammi, J. W. Ochterski, R. L. Martin, K. Morokuma, O. Farkas, J. B. Foresman, D. J. Fox, Wallingford, CT, **2016**; b) J. P. Perdew, K. Burke, M. Ernzerhof, *Phys. Rev. Lett.* **1996**, *77*, 3865-3868. doi:10.1103/PhysRevLett.77.3865

[S3] a) F. Weigend, R. Ahlrichs, *Phys. Chem. Chem. Phys.* **2005**, *7*, 3297-3305. doi:10.1039/B508541A; b) F. Weigend, *Phys. Chem. Chem. Phys.* **2006**, *8*, 1057-1065. doi:10.1039/B515623H

[S4] R. Herges, D. Geuenich, *J. Phys. Chem. A* **2001**, *105*, 3214-3220. doi:10.1021/jp0034426

[S5] a) J. P. Foster, F. Weinhold, *J. Am. Chem. Soc.* **1980**, *102*, 7211-7218. doi:10.1021/ja00544a007; b) A. E. Reed, L. A. Curtiss, F. Weinhold, *Chem. Rev.* **1988**, *88*, 899-926. doi:10.1021/cr00088a005

[S6] C. Lefrou, R. Cornut, *ChemPhysChem* **2010**, *11*, 547-556. doi:10.1002/cphc.200900600

[S7] J. M. Bernal-García, A. Guzmán-López, A. Cabrales-Torres, A. Estrada-Baltazar, G. A. Iglesias-Silva, *J. Chem. Eng. Data* **2008**, *53*, 1024-1027. doi:10.1021/je700671t

[S8] T. Jarusuwannapoom, W. Hongrojjanawiwat, S. Jitjaicham, L. Wannatong, M. Nithitanakul, C. Pattamaprom, P. Koombhongse, R. Rangkupan, P. Supaphol, *Eur. Polym. J.* **2005**, *41*, 409-421. doi:10.1016/j.eurpolymj.2004.10.010

[S9] P. K. Muhuri, D. K. Hazra, *J. Chem. Eng. Data* **1994**, *39*, 375-377. doi:10.1021/je00014a041

[S10] Y. Chernyak, *J. Chem. Eng. Data* **2006**, *51*, 416-418. doi:10.1021/je050341y

[S11] L. Mussari, M. Postigo, C. Lafuente, F. M. Royo, J. S. Urieta, *J. Chem. Eng. Data* **2000**, *45*, 86-91. doi:10.1021/je990211n

[S12] K. Müllen, H. Unterberg, W. Huber, O. Wennerström, U. Norinder, D. Tanner, B. Thulin, *J. Am. Chem. Soc.* **1984**, *106*, 7514-7522. doi:10.1021/ja00336a035

[S13] a) B. AXS, Madison, WI, **1998**, pp. SHELXTL v5.1, Bruker AXS; b) G. M. Sheldrick, *Acta Crystallogr.* **2015**, *C71*, 3-8. doi:10.1107/S2053229614024218
